# Paul Hertz’ contribution to proof theory: normal-form results for deductions

**DOI:** 10.1007/s00407-026-00377-9

**Published:** 2026-09-28

**Authors:** Moritz Bodner

**Affiliations:** Universität Wien, Vienna, Austria

## Abstract

Amongst the most central results in proof theory are normal-form results for deductions. Such results were first presented, not by David Hilbert, who first formulated the idea of a mathematical theory of mathematical proofs (in 1904), but by Paul Hertz (in 1922). Hertz’ work (and relationship to Hilbert) has not received the attention it deserves. I discuss both in detail, as well as the theoretical role normal-form results play within proof theory. An unpublished manuscript by Heinrich Behmann commenting on Hertz’ work is published (with translation) as an appendix.

## Introduction

“Proofs must themselves be treated as mathematical objects.” , David Hilbert asserted in 1904 and, with this as an animating idea, set out to create, more or less single-handedly, a new discipline he eventually called “proof theory”.^1^

The ambition expressed in this slogan, and consequently the object of proof theory, appears clear: it calls for the development of a mathematical theory of proofs in the same sense as there is a theory of, say, numbers, equations or curves. But dwell on this analogy for a moment. Recall that different mathematicians conceptualised (e.g.) curves differently: contrast, for instance, the synthetic geometry of Steiner and von Staudt with the, roughly contemporaneous development of analytic geometry by Plücker and Clebsch.^2^ So, to call for the development of a mathematical theory of something still leaves open which kind of theory—based on which concepts, using which methods, pursuing which results—is to be developed; i.e. what precisely the nature of its objects is.

This general lesson applies, in particular, to the case of a mathematical theory of mathematical proofs. I aim to show in this article that another protagonist in the early development of proof theory, Paul Hertz, conceived of it, already in Hilbert’s own time, unlike Hilbert did: Hilbert conceived of proofs primarily as what might be called “markers of provability” (because for him the main task of proof theory lay in establishing consistency, i.e. un-provability).^3^ Hertz, on the other hand, studied proofs as objects possessing a distinctive and intrinsically interesting syntactic structure in how their steps were arranged (because he developed proof theory in pursuit of independence results, which required him to investigate not only whether certain lines were generally (in)admissible, but also whether they were (in)dispensable where they occurred in a given proof, and its conclusion therefore (in)dependent of the claim made in that line).

Though Hilbert eventually recognised the relevance of studying (and manipulating) the syntax of deductions also for his purposes, he seems never to have come to regard it as intrinsically interesting; it remained for him, rather, a necessary preliminary to the proper purpose of proof theory, which consisted for Hilbert always mainly and only in establishing that no proof—be its structure what it may—could arrive at an end-formula which, like ‘0≠0’, indicated the inconsistency of the axiom system from which the proof takes its assumptions.

Speaking of different approaches gives rise to the temptation of describing the situation in general philosophical terms as a confrontation of different “paradigms”—proof theory on one hand as “provability theory”, as structural proof theory on the other—in the sense of Kuhn; one can speak of “paradigms” in this connexion, however, only in a looser sense, not least of all because, far from being incommensurable, work carried out in line with either one benefited also the other approach; the work of Hilbert, as well as Hertz’, each offers a ‘paradigm example’ of how to go about studying proofs as mathematical objects, but these approaches are not hermetically sealed off one from the other.

Another general conception seems to me to furnish a much more fitting description, namely, to describe (Hilbert’s) proof theory as a “research programme” in the sense of Lakatos:^4^ It has a patent “hard core”, which is shared: “Proofs must themselves be treated as mathematical objects.”^5^ Hilbert and Hertz differ by adhering to different “positive heuristics”, namely to establish consistency and independence respectively; in detail (see footnotes 39 and 67), by showing (respectively) that proofs cannot have as a last line a formula symptomatic of inconsistency, and that (only) certain deductions are trustworthy indicators of deductive dependence. Finally, between its inception in 1904 and (say) Gentzen’s first consistency proof for arithmetic with induction in Gentzen 1936, it goes through recognisable “positive problemshifts” (see footnote 40).

This article examines the relation between Hertz’ proof-theoretic work and Hilbert’s. After presenting biographical information about Hertz (in Sect. 2), I discuss, first (in Sect. 3), where Hertz took inspiration for his proof-theoretic work from Hilbert’s early programmatic remarks concerning a mathematical theory of mathematical proofs. Then I discuss (in Sect. 4) how Hertz’ proof-theoretic project differs from the proof theory Hilbert himself developed in the early 1920s, and show (in Sect. 4.2) that Hilbert almost certainly knew of Hertz’ technical work in some detail at a relatively early stage in his work on proof theory; in support of this claim, notes for a presentation to the Mathematical Colloquium in Göttingen by Heinrich Behmann, dealing with Hertz’ work in some depth, are discussed in Sect. 5, and published (with a facing English translation) as an appendix to this article. Finally, I argue (in Sect. 6) that it was precisely the divergence between their projects, which led to Hertz’ interest in normal-form results for entire deductions (all but ignored by Hilbert, who established only a very rudimentary such result and regarded it as a mere preliminary to proof theory proper)—which heralded the next decisive advance in proof theory—in the guise of Gentzen’s “Cut Elimination Theorem”—beyond anything Hilbert or his collaborators envisioned or accomplished (see Sect. 7).

## The life and work of Paul Hertz

Paul Hertz (1881–1940) was a physicist by training, and of some renown at the time.^6^ So, for example, Hertz’ work on thermodynamics was called “pristine” and “clear as light” by none other than Einstein,^7^ who, in a letter of recommendation written on Hertz’ behalf in 1922, maintained furthermore:

Mr. Hertz is not only a talented theoretical physicist but also a man of rare general education and outstanding critical talent. Einstein 2013, p. 265 (to Heinrich J. Goldschmidt, 17/IV/1922)

whilst calling Hertz in another such letter

without a doubt one of the most perceptive theoretical physicists in Germany, with a great knowledge of the field and remarkable originality. (dated 18/V/1922; 311 Einsten 2013, p. 311)

Over time Hertz drifted steadily away from physics towards philosophy, where, as he told Moritz Schlick in a letter dated 12/IX/1920, Hertz felt that he had found his true calling. So Hertz became a “physicist and philosopher”, as Bernays put it in the short biographical note mentioned in footnote 6; indeed, Hertz’ widow, Helene, thanked Bernays in a letter (dated 11/XI/1966, ETH, HA, Hs 975:2152) for describing her late husband in these terms since that was, she recalls, how Paul Hertz came ultimately to see himself.

Hertz’ work on proof theory falls mid-way along this transition.

Hertz received his doctorate (for work in physics) from the University of Göttingen in 1904,^8^ and returned there in 1912 to teach as a lecturer (Privatdozent) until he was appointed to a permanent position (außerordentlicher Professor) in 1921.^9^ The years between 1904–1912 Hertz spent, as he reports in a cv (see footnote 9), in “Paris, Göttingen, Berlin and Tübingen”, and the Netherlands, where he went “in the summer of 1911” to work with H. A. Lorentz.^10^ In 1909 Hertz obtained his licence to teach at university level in Heidelberg, again with work in physics,^11^ where he taught—except for the summer-semester of 1911, which he spent in Leiden with Lorentz—until his move back to Göttingen.

In the early 1920s, Hertz published two technical articles in the *Mathematische Annalen* treating certain meta-mathematical questions using what would nowadays be considered methods of (rudimentary) structural proof theory:

- “über Axiomensysteme für beliebige Satzsysteme. I. Teil” (1922)^12^
- “über Axiomensysteme für beliebige Satzsysteme. Teil II” (1923)^13^

In fact, Hertz had presented an outline of his project already in 1921 at the annual meeting of the German Mathematical Association. The text of this presentation, intended for a non-specialist audience, was also published as

- Axiomensysteme kleinster Satzzahl (1921)

It is worth noting that, according to the preliminary programme for the “Annual Meeting in Jena”,^14^ Hertz gave his presentation on 22/IX/1921 in the same session as Bernays (who presented on “Hilbert’s work concerning the foundations of arithmetic”), Fraenkel and Hahn. That Bernays presented immediately before Hertz, raises the distinct possibility that Hilbert learned about the contents of Hertz’ presentation from Bernays.^15^

These publications were followed, about half a decade later, in 1929, by another substantive technical article in the *Mathematische Annalen*, where Hertz presented, first, some of the ideas and results already presented in his earlier articles, and then developed them further and introduced some new ideas:

- “Über Axiomensysteme für beliebige Satzsysteme [III]” (1929)

This article was also accompanied by another non-technical exposition of his results published the same year, bearing an only marginally different title:

- “Über Axiomensysteme beliebiger Satzsysteme” (1929)

Although Hertz’ technical work was *published* as late as 1929, it was likely substantially completed as early as 1924.^16^ There is no indication that Hertz did any further technical work in proof theory after the mid-1920s.

When Hertz subsequently mentions new work in logic in correspondence with Bernays (in a postscript to the letter dated 30/III/1937 (ETH, HA Hs 975:2146) and in the letter dated 12/V/1938 (ETH, HA Hs 975:2147)), he apparently means further developments of the philosophically oriented writings on logic he published in the late 1920s and early 1930s, not any new technical work.

One possible reason why Hertz appears to have effectively abandoned his proof-theoretical work in the mid-1920s is suggested by Hertz’ own later assessment of the prospects of his approach: Hertz’ himself understood that his methods fell short of their goal but had no further ideas for improving them; so, Hertz 1929b, p. 458 merely expresses the hope that “our problem might be solved by means of improved methods” (cf. Hertz 1929a, p. 195).

Besides his proof-theoretic work, Hertz also kept up his work on physics, and turned, in the course of time, increasingly towards philosophy.^17^

Indeed, Hertz’ personnel file kept at the university archive of the university of Göttingen shows that Hertz made efforts to align his position with his shifting interests in the later 1920s, petitioning (in a letter dated 10/VI/1926) for an extension of his licence to teach, granted originally only for physics and “the methodology of the exact sciences”^18^ to cover also philosophy more broadly.

Although these efforts eventually succeeded—albeit after years of “opposition” (mentioned but not described in specific detail in Hertz’ personnel file)—and Hertz’ licence to teach was finally extended to “logic, theory of knowledge and natural philosophy” (as a letter dated 27/VI/1932 attests), this was to be of little use because Hertz’ licence to teach was revoked altogether by the Nazi government a year later on 14/IX/1933, and Hertz forced into emigration.

Altogether Hertz’ academic career was not crowned by success. The reasons for this are diverse and manifold: In the first instance the vagrancies of the post-WWI era in the Weimar Republic did not treat Hertz kindly. As a mere (unsalaried) lecturer and (later) extraordinary professor (with a meagre stipend (initially) or salary (eventually)),^19^ Hertz struggled to make ends meet, forcing him to divert time and attention away from his academic work to provide for his wife and three children.^20^ In fact, in 1920, Hertz appears to have considered (as a letter to Einstein, dated 11/XI/1920, (Einstein 2006, pp. 487f.), pp. 487 sq.), indicates) leaving academia altogether to work in a patent office or as a mathematician for the company Siemens-Schuckert, but, as he tells Einstein, Hertz “couldn’t in the end resolve to just detach myself from purely theoretical work [...] so I will try to see if it is possible to stay in Göttingen”.^21^

That Hertz did not contribute decisively to the ground-breaking advances in theoretical physics taking place during his lifetime^22^, and that, moreover, the focus of Hertz’ research shifted steadily away from physics altogether, towards philosophy, also entailed that Hertz never stood as the obvious candidate for top-tier academic employment^23^ either in philosophy (where he was not established enough) or in physics (with which he was increasingly out of touch),^24^ in spite of the fact that Hertz was, according to reports, an excellent lecturer.^25^

The precarious circumstances in which Hertz therefore spent much of his life may, in turn, have been one main reason why almost all of Hertz’ projects appear to have stalled or appeared in the end as little more than shadows of his ambitions:^26^ Hertz’ only published philosophical book, *Über das Denken* (Hertz 1923b), bears the subtitle “First Part”, but no further part was ever published. (Hertz did publish one other book, Hertz 1916: a concise introduction to statistical mechanics; amongst Hertz’ papers there exists furthermore a bound (book length) volume (under the signature “PH 03-03”) of polished lecture notes also covering statistical mechanics, as well as a volume of lecture notes (from 1913) covering “Strahlungs- und Quantentheorie” (“PH 03-02”).)

During the last 15 years of his life, Hertz was apparently working on a monograph on the philosophy of logic—perhaps originally begun and intended as the second part to his 1923 monograph. Hertz himself mentions “my not yet published book on the foundations of logic” in his petition to have his licence to teach extended to philosophy (see p. 6), and expresses the hope “to submit [in support of his petition] the manuscript in whole or in part in a couple of months at the earliest”. In the end, it remained unpublished altogether. Amongst Hertz’ papers, kept by the Archive of Scientific Philosophy in Pittsburgh, two (incomplete) drafts of book length are found dealing with the philosophy of logic: One, comprising a total of 305 typewritten pages under the signatures “PH 03-03” (first half) and “PH 03-04” (second half). Another, comprising 135 typewritten pages, under the signature “PH 03-06”. These typescripts presumably represent a substantive portion, if not all of Hertz’ book on the philosophy of logic. The existence of two extensive, yet incomplete drafts, each substantially different in form and content, suggests that, until the end of his working life, Hertz could not complete (even a full outline of) the book to his satisfaction. Schlick, indeed, after knowing and maintaining a friendly relationship with Hertz for more than a decade, wrote to Philipp Frank (in a letter dated 2/V/1931) that he thought Hertz unlikely to ever produce a viable manuscript for his monograph on logic.

Unkind circumstances were not the only reason why Hertz’ work remained unfinished: Hertz seems to have regularly, seemingly habitually, started new projects only to abandon them later.^27^ Indeed, Hertz’ work on proof theory gives another example of such a project Hertz’ devoted himself to for some time, before abandoning it again, relatively soon after starting.^28^ In the letter to Bernays dated 30/III/1937 (ETH, HA Hs. 975:2146), Hertz himself explains the lack of progress in his work by observing how each of his projects invariably seems to get displaced by another, without being at all finished.

Hertz also seems to have routinely underestimated the kind and the amount of work required to carry out his projects even to his own satisfaction, which perhaps caused him to become discouraged and abandon them or let them be displaced by other, new projects. In his correspondence with Schlick about the project that eventually became Hertz’ 1923 monograph, *Über das Denken,* Hertz reports (in a letter dated 20/IV/1920) his discouragement upon discovering, seemingly to his surprise, how much had already been written on the topic by psychologists, whose work Hertz now felt obliged to take into consideration; in a subsequent letter, dated 30/IV/1920, Hertz reports that he managed to largely overcome this discouragement. Concerning Hertz’ work on the philosophy of logic, Carnap records in his diary his “reservations about publishing” it (Carnap 2022, p. 471, (entry for 13/VI/1930)) and later (p. 721, entry for 21/XI/1935) his estimation that “[Hertz] gets caught up in pseudo-questions sometimes”.^29^

In the 1930s Hertz, who was Jewish, fell victim to the Nazi regime’s purge of academic institutions. He was forced, after briefly holding a professorship for physics in Prague, to emigrate and, after drifting from post to post for almost a decade, died in Philadelphia on 24/III/1940. His son, Rudolf, writes in his biographical sketch (referred to in footnote 7, p. 11) that his father “arrived in America [in 1938], no overcoat and ten marks in his pocket. He was a broken man, physically and mentally”. What remained of Hertz’ papers (lecture notes, offprints, unpublished manuscripts and correspondence) was given by his son, Rudolf, to the Archive of Scientific Philosophy in Pittsburgh.^30^

## Hertz’ proof-theoretic project (I): concepts

Already in his presentation of 1921, Hertz stated clearly that his work concerned another one of the three main meta-theoretic problems Hilbert had raised (see, e.g. the official lecture notes for his 1921/22 course: Hilbert 2013, p. 435.). Hertz focussed not (as Hilbert did) on consistency, nor—the last problem Hilbert stated in his programmatic discussions of the axiomatic method^31^—their completeness, but rather on the independence of axioms. So Hertz began:

The choice of independent axioms is to a certain extent left to arbitrariness. One can ask now in which cases it is possible only in *one* way, and, in cases where there are multiple systems of independent axioms, how to find that one which comprises the least number of sentences. Concerning this task some notes shall be communicated in what follows.^32^

In pursuit of this programme, Hertz aimed, on the one hand, to find a criterion for ascertaining the independence of what he calls “sentences” (“Sätze” ) and, on the other hand, a method for isolating from a given axiom system, some of whose axioms are *inter*-dependent (given the other axioms of the system), an *in*-dependent axiom system from which all the same consequences are deducible.

### Hertz’ conception of “sentences”

I want to emphasise at this point that what Hertz called a “Satz” is not what would nowadays be called a “sentence” in the sense of “closed well-formed formula in a particular artificial (first-order) language $\mathcal {L}$”; rather, a sentence in Hertz’ sense is more closely related to what would nowadays be called a “sequent”,^33^ i.e. a (meta-theoretic) statement expressing the deducibility of a proposition (in the sense of “well-formed formula in the language $\mathcal {L}$”^34^) from one or several other such statements, though the way Hertz himself presents what he calls “sentences” casts them as expressions of a more general, abstract relation of dependence, which comprises as special cases expressions of deductive dependence amongst statements in a language—in which case sentences are akin to sequents—as well as expressions of dependence amongst physical events, for instance (see Legris 2014, p. 95 for some discussion). Hertz remarks furthermore in a footnote that “our sentences a→b are nothing other than formal “implications” in the sense of Russell”.^35^ This remark is at loggerheads with Hertz’ more abstract example insofar as it is clear *in Russell’s exposition* that formal implications obtain only between propositions; in fact, Russell 1937, p. 15 characterises propositions precisely as things fit to be component of formal implications.^36^

Of course, Hertz’ ‘formal’ methods are such that what their inventor had in mind does not prevent their application in other cases, specifically to closed formulas of artificial first-order languages as nowadays standardly conceived; a point made explicitly by Gentzen 1932, p. 330, who noted in his exposition of Hertz’ ideas that “we care only about the formal shape [of a sentence]”.

Still, highlighting that “sentence” in Hertz’ usage does not mean “(closed) well-formed formula” is significant for two reasons, one theoretical, the other historical: Theoretically, the divergence of Hertz’ usage of “sentence” from what might for readers now be the default sense of this word highlights a consequent divergence of Hertz’ project from anything that is nowadays pursued.

When Hertz says that he is concerned with the independence of axioms for systems of sentences, “sentence” must be understood in Hertz’ sense: Hertz is accordingly, in effect, concerned with the independence of *propositions expressing dependencies* between abstract elements, not with the independence amongst the elements themselves. In the specific instance where the elements are statements in a language, this amounts to saying that Hertz is not interested in the deductive (in-)dependence amongst these statements themselves, but rather in the dependence *amongst propositions expressing deductive dependence* amongst statements. Hertz is, in other words, concerned with (meta-theoretically) *systematising* the *relationships of dependence amongst* given abstract elements, not with (object-theoretically) ascertaining such relationships in particular cases (by ‘deriving’ (in an abstract sense) one *element* from (some) other(s)).^37^ The aim of this systematisation consists in deriving all of these (meta-theoretic) propositions expressing dependence amongst elements can be derived from a minimal selection of base dependence expressions—which Hertz calls “axioms” (in a suitably abstract sense corresponding to the abstract sense of “sentence”)—by means of two rules of procedure to derive dependencies from dependencies.^38^

By contrast, when a logician nowadays speaks about the “independence of sentences”, the concern is usually with independence between (object-theoretic) (closed) well-formed formulas, not between (meta-theoretic) assertions of deductive dependency amongst them; in other words, logicians today are concerned with the assertion of individual claims about deductive (in)dependence, not with the derivability of such dependence claims from one another. In fact, the axiomatisation of meta-theoretic assertions is rarely thematised at all.

### Hertz’ and Hilbert’s proof-theoretical programme

Hilbert, too, in his mature writings about the axiomatic method formulates his triad of problems—consistency, independence and completeness—with respect to object-theoretic well-formed formulas (e.g. in Hilbert 1935d, p. 161): After Hilbert introduced precisely defined (artificial) languages (in 1917/18), developed axiomatisations of sentential and first-order logic, he revisited his ideas for a mathematical theory of proofs from 1904. The technical details became greatly clarified as a result, whilst progress on the central issue—establishing the consistency of an axiom system by means other than exhibiting a model with a domain of pre-existing mathematical objects which (in specific constellations) satisfy all the axioms^39^—remained more or less as it was in 1904. In 1921, the problem of establishing the consistency of (now precisely formulated) axiom systems finally takes centre-stage in Hilbert’s mind. Hilbert goes so far as to claim that all other problems which routinely arise in applications of the axiomatic method are “subordinate to the aspect of consistency” (so Hilbert says in the introductory remarks for his 1921/22 course, see Hilbert 2013, p. 435) because, as a marginal note Hilbert added in the official lecture notes clarifies: the answer to the proof-theoretic questions of provability and consistency “*ipso facto* constitute the development of the theory and contain everything that is essential!” (this marginal note is reported in Hilbert 2013, p. 496).

This is how mathematics progresses, according to Hilbert 1935b, p. 180:

in two ways in constant alternation: by obtaining new provable formulas from the axioms by means of formalised inferences [formales Schließen]; on the other hand by adding new axioms^40^ and establishing their consistency by means of intuitive inferences [inhaltliches Schließen].

With the lectures in 1922/23, Hilbert’s proof theory reached maturity also in the sense that he does not develop it substantially further. Hilbert shifted his attention instead onto other matters—such as his popular lectures on the infinite, and his work on synthetic geometry (with Cohn-Vossen)—leaving proof theory in the (capable) hands of his students and collaborators.

However, as the qualification “mature” was meant to foreshadow, and this brief sketch of Hilbert’s development detailed to some extent, Hilbert himself did not always draw the basic technical distinctions of importance in proof theory clearly and, as I pointed out (in footnote 42) , Hilbert’s earliest writings about proof theory and logic suggest a setting for these same ideas and problems more in line with Hertz’ writings. This is, as a matter of fact, no accident.

This is the other reason why the divergence of Hertz’ usage of “sentence” from what has since become standard is of interest: because this divergence shows how Hertz’ work could have grown out of Hilbert’s earlier writings, in particular, out of Hilbert’s first sketch for a mathematical theory of proofs.^41^

Already in Hertz’ abstract description of sentences as consisting of “connected elements” (see footnote 34) an echo of an odd remark from Hilbert’s initial sketch can be discerned: Hilbert 1905, p. 185 describes a proof there in abstract terms as “a mathematical object, specifically a finite set [...] whose elements are connected by statements [“Aussagen”] which express that the proofs leads from the [premisses] to the [conclusions]” . What Hertz in 1921 calls “sentences” (“Sätze”) are precisely such statements of deductive dependence which Hilbert in this quotation calls “statements” (“Aussagen”) .^42^

Hertz’ explanation of what he calls “sentences” in Hertz (1922) brings into focus additional similarities to Hilbert 1905. Hertz 1922, pp. 246 sq.writes:

We consider here only sentences of a certain type, sentences which we symbolically write: $(a_1,\dots ,a_n)\rightarrow b$ and which we express linguistically by way of formulations like the following: If $(a_1,\dots , a_n)$ taken together are, then *b* is. [...] | In addition, we accept rules, according to which from certain sentences others follow: So, for example, the validity of the sentences a→b, b→c shall entail the obtaining of the sentence a→c.

This explanation echoes Hilbert’s sketch in three respects:

(i) Hertz takes compound antecedents of sentences $(a_1,\dots , a_n)$ to be conjunctions by default, as Hilbert 1905, p. 177 did;^43^
(ii) the component parts $a_i$ and *b* of sentences are said to “be”—rather than “be true”—as was Hilbert’s way of speaking about what he called “combinations” in 1904 (as belonging in the “class of beings” or “nonbeings” respectively. So Hertz’ appears to use not only the word “sentence” like Hilbert used the word “statement” (as I pointed out on p. 12), but also to use “elements” like Hilbert used the word “combination”: for the (abstract) components of sentences/statements; and
(iii) the main inference rule Hertz accepts is the same—although Hertz in the above passage only states a particular case: of sentences that have only a single antecedent—as the transitivity rule for conditional assertions Hilbert relied on in Hilbert 1905, p. 178. A reviewer has pointed out that the rule also appears, closer to 1920, in Bernays’ Habilitationsschrift (as “Regel d)”, see Hilbert 2013, p. 245), to which Hertz would have had access in Göttingen. However, that Hertz does explicitly mention Bernays later (see Sect. 4.1), but not at first, suggests to my mind that Hertz did not discuss his work with Bernays initially, and may not have been aware of Bernays’ work at that point.

Although Hilbert’s early sketches for a proof theory provided important impulses, Hertz’ work remained by no means confined to merely implementing Hilbert’s ideas. I will in the following sections highlight two ways in which Hertz took decisive steps well beyond anything Hilbert had envisioned (in 1904), and towards what eventually became structural proof theory: Hertz 1923a introduced a concept of deduction which requires, unlike Hilbert’s (which is now standard), that the arrangement of the lines be ‘disentangled’ and made explicit (Hertz’ calls this an “inference system” ) and discusses at some length how it relates to something approximately like deductions in Hilbert’s sense. In keeping with this emphasis on an orderly arrangement of steps, in Hertz’ work normal-form results for deductions—on which I will focus in Sect. 6—play a central role , as they do subsequently for Gentzen (see Sect. 7).

### Hertz’ concept of an inference system

In his second technical article, Hertz (1923a) discusses two precisely defined notions of deduction—one Hertz calls “proof” (“Beweis”), the other “inference system” (“Schlusssystem”)—and discusses their interrelation. What Hertz calls “proofs” are linear sequences of inferences arranged in such a way that all inferences which have the premisses of a given inference as conclusions must come *before* the inference invoking those premisses, though they need not come always *immediately before* the conclusions depending on them (the definition of proof is given in passing in art. 26 of Hertz 1923a, p. 84).^44^ In fact, the arrangement of inferences supplying the premisses of subsequent inferences is arbitrary. (In substance, if not in formulation, this is the now standard conception of ‘cascading’, “Hilbert-style” linear proofs.)

Hertz also introduces the word “chain inference” (“Kettenschluss”), defined (in art. 23) as “a simple ordered sequence of finitely many inferences with the property that every non-tautological premiss [i.e., not of the form $\mathfrak {a}\rightarrow \mathfrak {a}$, see art. 7 (p. 81)] which does not belong to the [premiss set] $\mathfrak {T}$ coincides with an earlier [but *not* necessarily immediately preceding] conclusion”, as near synonymous with “proof”: The difference between “inference chain” and “proof” is, as far as I can tell, minor, namely Hertz uses “proof” only in connexion with a specific conclusion to say that a certain inference chain is a “proof of $\mathfrak {a}$”, whilst “inference chain” is used (also) in contexts when no particular conclusion is specified.

By contrast to such proofs (or chain inferences) in Hertz’ terminological sense, in what Hertz calls “inference system” (defined in art. 38, Hertz 1923a, p. 86) lines are arranged in a completely fixed order, such that every conclusion stands in this order *immediately* after the premiss(es) from which it is deduced. To be precise, Hertz defines an inference system as a specific kind of what he calls an “inference aggregate” (defined in art. 35, Hertz 1923a, pp. 85 sq.) as “a system $\mathfrak {U}$ of finitely many inferences either in itself or with reference to a relation $\textsf{Z}$ between premisses and conclusions by which a conclusion is related to at most one premiss of another inference which has the same syntactical form”. The relation “$\textsf{Z}$” thus fixes the (non-linear) arrangement of the inferences.

An inference system (as defined in Hertz’ art. 38) is an inference aggregate where $\textsf{Z}$ is well-founded, i.e. where $\textsf{Z}$ does not relate premisses to conclusions in a circular fashion. This ordering relation $\textsf{Z}$ need not be linear—and is, in fact, generally (as in Gentzen-style proof trees): non-linear. Otherwise no conclusion could depend on more than one premiss, since nothing can have two (or more) *immediate* predecessors in a *linear* order; but Hertz’ main rule of inference does indeed require two premisses, so from each application of this rule two parallel branches grow upwards towards, ultimately, axioms:

$$\begin{aligned} \begin{array}{c}\underbrace{\begin{array}{cc}a\rightarrow b& b\rightarrow c\end{array}}\\ a\rightarrow c\end{array} \end{aligned}$$

Hertz calls this rule—effectively a transitivity rule for ‘→’—“syllogism”.^45^

Hertz finally defines what he calls a “primitive inference system” by the additional condition that it has a single bottom-most conclusion, or, in Hertz’ technical terms: as an inference system where at least one conclusion is not related by $\textsf{Z}$ to any premiss of a further inference (see arts 46 and 45, Hertz 1923a, p. 87). This is, in effect, the kind of constellation Gentzen’s trees visualise.

The main result Hertz establishes concerning proofs and inference systems is that a primitive inference system with a given sentence as conclusion can be found always and only in case there is a proof of that sentence. Hertz states the biconditional in art. 50, referring back to arts. 40 (Hertz 1923a, pp. 86 sq.) and 49 (Hertz 1923a, p. 88), which establish, respectively, that the existence of a proof is sufficient for the existence of an inference system and also necessary.

Hertz’ argument to establish this result resembles Hilbert’s argument to establish that lemma of his mature proof theory which comes closest to qualifying as a piece of structural proof theory within Hilbert’s otherwise not structurally minded approach:^46^ Given a “proof” in Hertz’ technical sense, one obtains what Hertz calls an inference system—if any change is needed at all—by “multiplying as needed” certain inferences, effectively ‘branching’ the linear order of the proof so as to make it satisfy the definitional requirement that it must assign any given conclusion to *at most one* subsequent premiss. Conversely, given a (non-primitive) inference system, a proof is obtained by ‘linearising’ the order-relation $\textsf{Z}$ so that any sentences which are equally far removed by $\textsf{Z}$ from a given sentence are arranged any which way with respect to one another.

To each of these processes corresponds a like process in the procedure described by Hilbert’s Distentanglement Lemma:^47^ the duplication, where necessary, of lines until every line (apart from the ultimate conclusion) is cited as a premiss only once,^48^ and the excision of lines which are never used as premisses.

According to the student notes of Hilbert’s 1922/23 lectures, Hilbert, like Hertz, introduced in this context a piece of relational terminology in the lecture of 14/XII/1922 (see Hilbert 2013, p. 615) calling a conclusion the “issue” (“Kömmling”^49^) of the earlier conclusions (or axioms) serving as the premisses of the application of the inference rule by which it is derived. In the official notes for his 1921/22 lecture course the expression “motivating formula” is used instead (Hilbert 2013, pp. 506 sq.). That “motivating formula” plays the same role as “issue” a marginal note (reported on Hilbert 2013, p. 525) makes clear, wherein Hilbert added “des Kömmlings” as an alternative to “der motivierenden Formel” in the phrase “den Begriff der “*motivierenden Formel*” einführen”; the only difference is one of perspective: “issue” applies to the later line, *which* ‘issues’ from earlier lines, whilst “motivating formula” applies to those earlier lines which ‘motivate’ a later line; Hilbert 1935b, p. 184 contains a hybrid formulation, speaking of every formula “having an issue, which it serves to motivate”. Although Hilbert speaks of “issue” or “motivating formula” without explicitly introducing a relation like Hertz’ $\textsf{Z}$ between conclusions and premisses, such a relation is clearly implicit in Hilbert’s exposition, since Hilbert speaks of “relating” each formula to its motivating formula—using the same German verb, “zuordnen” (Hilbert 2013, p. 507), which Hertz 1923a, 86 uses also.

Hilbert 2013, p. 507 formulates the Disentanglement Lemma using this relational terminology as follows: When the threads of a proof are disentangled, every conclusion has exactly one motivating formula.

## Relation between Hertz & Hilbert

In the light of the similarities between the effects of Hilbert’s Disentanglement Lemma and Hertz distinction between proofs and inference systems—and Hertz’ associate results about obtaining each from the other—one is bound to consider the question whether the idea seemingly expressed in the proof-theoretic work of both Hertz and Hilbert at almost the exact same time (in 1922) originated with one of them and was then adopted by the other. It is, of course, possible that both came up with it independently, working in comparable settings giving them starting points for analogous considerations (see Sect. 3.2).

However, seeing the perfunctory treatment of these ideas in Hilbert’s work,^50^ in contrast to the extensive and rigorous discussion in Hertz’ article—as, e.g. attest by Hertz’ explicit introduction of the relation $\textsf{Z}$, see p. 15 –, may be seen as a sign that Hilbert—or Bernays, who is reported to be lecturing on 14/XII/1922—gleaned these ideas and results from Hertz.

Influence in the converse direction is unlikely on purely chronological grounds: Hertz’ second technical article was received by the *Mathematische Annalen* on 2/VI/1922 (as recorded in Hertz 1923a, p. 102), before Hilbert first mentioned such results either in print (in: Hilbert 1935b, p. 184) or in lectures (on 14/XII/1922, see footnote 50). Hertz’ actual work must therefore have been finished before the summer of 1922—the completion of the manuscript is dated (as also recorded in Hertz 1923a, p. 102) to 20/V/1922, though that gives no clear indication of when the bulk of the technical work was done—when Hertz submitted what he regarded as sufficiently polished form of it for publication, thus at a time when Hilbert’s lectures still show no trace of such ideas.

The same chronological considerations support the hypothesis that Hilbert knew Hertz’ proof-theoretic work. Hilbert presumably saw it—assuming he didn’t know of it previously through conversations with Hertz (of which there is no record—but there rarely is of conversations, even those sure to have taken place)^51^—at the latest when it was submitted to the *Mathematische Annalen* (and would have seen Hertz’ first article submitted the year before and published in 1922). There is, in any case, solid evidence that Hilbert was exposed to Hertz’ work in some detail in November 1922—a few weeks before Hilbert first expounded such ideas in lectures—, which I will turn in section 4.2.

### Personal interactions

Bernays, in any case, certainly knew Hertz’ work by spring 1922 at the latest, and even discussed it with Hertz in person (as Hertz acknowledges, see footnote 50 and below); indeed, these discussions provide a plausible path for Hertz’ ideas to have entered Hilbert’s proof-theoretic work in 1922. Hertz acknowledges Bernays’ input at various points (see: Hertz 1923a, p. 81 and Hertz 1929b, pp. 475fn32, 479, 482fn38); and Bernays, in turn, writes in a letter of condolence to Hertz’ widow (dated only “April 1940”, ETH, HA Hs 975:2148a) that conversations with Hertz afforded him “much valuable stimulation, and also my philosophical views were influenced by him in many ways”.

There are no traces of similar personal interactions discussing proof theory with Hilbert; Hertz cites Hilbert’s published articles (see Hertz 1929b, p. 464 fn. 14, p. 502, fn. 51), but never mentions specific comments from, nor general conversations with Hilbert.^52^ About the reasons why Hilbert and Hertz seemingly never so much as spoke about proof theory one can only speculate.^53^

It seems all the more baffling in the light of a letter (dated 26/XII/1914) Hilbert wrote to Hugo Dingler (quoted by Mancosu 2010b, p. 514), where Hilbert explicitly mentions Hertz as one of several colleagues—describing them, as Mancosu 2010c, p. 178 does in paraphrasing the letter, as “[Hilbert’s] closest collaborators on foundational issues” strikes me as an overstatement, however, since there is no evidence that Hilbert ever worked closely with any of the men he mentions there (besides Hertz: Grelling and Bernstein)—who “are interested in the epistemological aspects of mathematics”.

This letter shows that Hilbert knew of Hertz’ interest in foundational matters—and, presumably, also of whatever foundational work Hertz was doing at the time—from early on; and yet, later, when Hertz did actual technical work on foundational issues, which even was very closely related to Hilbert’s own work at the time, Hilbert appears, so far as the historical record goes, to have taken no active or lasting interest in it.

I should emphasise at this point that even if Hilbert’s talk of “issue” in the context of proof theory and the Disentanglement Lemma are, in fact, traces of influence of Hertz’ work on Hilbert—for which, as I indicated on p. 9, in turn Hilbert’s earlier proof-theoretic sketch served as a point of departure—the impact of Hertz’ proof theory on Hilbert’s can still not be considered as anything more than marginal, seeing how Hilbert assigned to these ideas and results only ever a minor, preliminary role in his own version of proof theory .

Hertz’ work could, however, have had a tangible impact on Hilbert’s thinking on a more central, but less technical point: Already in his first programmatic article (see footnote 14), and again, in more detail, in §. 3 of his first technical article (see: Hertz Hertz (1922), p. 261–269), Hertz spoke of introducing “ideal elements” into systems of sentences. Hilbert likewise assigns a prominent methodological role to what he calls “ideal statements”, for the first time in his 1924/25 lecture course on the infinite (see, e.g.: Hilbert 2013, p. 751; cf. the introductions to these lectures, and the volume Hilbert 2013, p. 22, 658).^54^

The “first mention of the use of ideal elements as a general method in mathematics” occurs in Hilbert’s 1898–99 lecture course on Euclidean geometry (see Hilbert 2003, p. 249, especially editorial footnote 150). Hilbert discusses the method again at some length, citing also the example of ideal factors in number theory, in his 1919/20 lecture course (see: Hilbert 1992, pp. 94–100), so well before Hertz. However, the *transfer of these ideas* from geometry and number theory *to logic*—speaking not just of ideal elements (in the domain) to which general statements in a given theory are applicable, but of *ideal statements being part of the theory*—takes place only in Hilbert’s 1924/25 lecture course on the infinite, after (Hilbert’s encounter with) Hertz’ pertinent work.

As in the case of the Distentanglement Lemma, so in this case, there is the possibility that Hilbert and Hertz came up with the same idea independently, thinking about similar issues in the same environment and on the basis of shared starting points, hence ultimately along the same lines.^55^ Hilbert, for example, characterised completeness in 1917/18—before adopting the syntactic conception of completeness usually credited to Post c.f. Franks 2023, p. 283—in terms of the impossibility of consistently “adding [new formulas] to the system of base formulas” (Hilbert 2013, p. 157), which shows that Hilbert, like Hertz, occasionally viewed also entire theories as sets of sentences to which new elements might be added, as one adds new points (at infinity) to the set of points in geometry, or new (ideal) divisors to a number field.

But even if the idea of adding ideal propositions occurred to Hilbert and Hertz independently (and, again, it is clear for chronological reasons that Hertz is not adopting Hilbert’s ideas since Hertz articulated his own in print years before Hilbert did), it is remarkable just how similar their ideas turned out to be. Hertz 1921, p. 156 describes such ideal sentences as follows:

Such an ideal element serves the purpose of allowing for a convenient presentation of the sentences between real elements [viz. the deductive dependencies between object-theoretic claims]. The sentences between real and ideal elements or between [just] ideal elements have no meaning in themselves, rather only the sentences between ideal elements which are derived from them do [have meaning].

Like Hertz here, Hilbert later denies that what he calls “ideal statements” have “any meaning on their own” (see: Hilbert 1930a, p. 298). Like Hertz in his emphasis on convenience as a motivation for introducing ideal elements, Hilbert too motivates their introduction there by observing that, with them, one obtains “a system of propositions, in which the simple rules of Aristotelian logic are fully valid”; though for both Hertz and Hilbert the point is not *just* convenience, but also theoretical uniformity: for Hilbert of the applicability of logical rules, for Hertz of the axiomatisability of systems of sentences. Finally, Hilbert stresses, again like Hertz, that, even though ideal statements are meaningless, “still from them [real] statements can be deduced” (Hilbert 2013, p. 750; see also Hilbert 1930c, p. 281 in the published 1926 article condensed from these lectures).

That being said, a similar qualification is in order as before: Even if it was Hertz’ work that gave Hilbert the basic idea to introduce “ideal elements” also *amongst the statements* of an axiomatic theory, Hilbert figured out for himself the crucial details for himself, namely: how to classify which statements—that only purely numerical statements qualify as real, and all others are ideal.

Still, that Hertz’ first programmatic article not only prefigures—however vaguely—a prominent theme of Hilbert’s later work, but inspired it, is not altogether unlikely also because this article seems to have been the work of Hertz’ that was best known in Göttingen, as I document in the following section.

### The Göttinger “Hertz-Week” in 1922

In the fall of 1922, the Göttingen Mathematical Society devoted two weeks to Hertz’ proof-theoretic work, as reported in the *Jahresberichte der Deutschen Mathematiker-Vereinigung*.^56^ According to the report in the *JDM* (vol. 32, 1923), p. 21 of the part conveying “Mitteilungen und Nachrichten” (paginated separately from the main part of the *JDM* comprising research articles) Hertz himself presented “on axiom systems” to the Mathematische Gesellschaft in Göttingen on 21/XI/1922 (a few weeks before Hilbert’s lecture course came to the point of introducing what I’ve called the (possibly Hertz-inspired) “Diesentanglement Lemma”). At the next meeting, on 28/XI/1922, Emmy Noether followed up on Hertz’ presentation with “group-theoretic remarks concerning the problem of axiomatisation” and, as the *Jahresberichte* go on to report, observed that “the considerations of *P. Hertz* can be recaptured by means of group theory”^57^ (unfortunately no record of her presentation survives).^58^

On the same date, Heinrich Behmann is reported to have spoken on “the problem of axiomatisation from the point of view of the algebra of logic”. Although Behmann gave his presentation this seemingly very general title, his preparatory notes, which survive amongst Behmann’s papers, reveal that Behmann, like Emmy Noether before him, mainly discussed Hertz’ work, especially Hertz’ first programmatic article, and did so in considerable detail.^59^ Indeed, under that general title, Behmann’s notes begin by announcing simply: “*Remarks* [following up] on the presentation of H[ertz].”

By the standards of the Göttinger Mathematische Gesellschaft’s usual agenda, such as it is reported in the *JDM*, this concentration on a single author or topic for more than one session, was unusual: a lot of the sessions are dedicated to two (or more) totally different topics (e.g. in the session of 14/XI/1922 Bernstein spoke first about M. J. Keynes’ book on probability theory, then Kérekjártó spoke about the characteristic of surfaces and finally Kneser spoke about isotropy of closed surfaces; or on 5/XII/1922, Ore spoke first about irreducible algebraic equations, followed by a presentation by Courant on integral equations; or on 9/I/1923, Grandjot spoke first about recent results obtained by Hardy & Littlewood, then Bernays presented on Skolem’s “Logisch-kombinatorische Untersuchungen über Erfüllbarkeit oder Beweisbarkeit mathematischer Sätze” [!]). What led to the unusual concentration of attention on Hertz’ work in November 1922, why what I’ve tendentiously called the “Hertz-Week” occurred—whether at Hertz’ behest, or perhaps upon Hilbert’s request, or maybe following Bernays’ initiative, or whatever other reason—is not documented. But the very fact that they occurred attests that mathematicians in Göttingen, including some of those close to Hilbert—like Behmann or Emmy Noether, and, of course, Bernays (as I discussed on p. 18) –, saw in Hertz’ work something “novel and noteworthy”, as Behmann said in his presentation.

Ultimately Behmann’s estimation of the sum total of Hertz’ efforts is less enthusiastic than his initial assessment that Hertz raised a “new and noteworthy problem” and suggested “suitable” methods to approach it. Behmann goes on to observe that the applications Hertz presented of his concepts and methods were “utterly *trivial*” (Behmann’s emphasis), owing to the fact that there is a decision procedure for the kinds of sentences Hertz considers.^60^ Hence, Behmann maintains, for the kind of inferences Hertz had so far studied “the heavy weaponry of general proof theory is on this level not yet appropriate”.^61^

In fact, I think Behmann’s assessment should be qualified in the light of the reasoning underpinning it: Behmann maintains that for the simplistic setting Hertz had confined himself to—effectively something like a (fragment of) sentential logic—one has no need for procedures to transform deductions since the (available) decision procedure, in a sense, provides a canonical kind of proof for any proposition (see p. 40). However interesting Behmann’s remark is in its own right, using it as a reason to denigrate Hertz’ methods seems facetious: Behmann seems set throughout his presentation on showing the superiority of his own approach to logic, to which the decision problem was central: Behmann wants to demonstrate that it can do what Hertz’ approach could do, and more. Right as that may be, it does not imply that Hertz’ approach is of any less interest. One might note, for instance, that it is still interesting to compare other (non-canonical) proofs of the same proposition to the canonical proof supplied by the decision procedure, as well as to one another, and comparing their respective normal forms provides one way of doing so (cf.: Prawitz 1971, p. 257). The intrinsic interest of investigating the structure of proofs Behmann seems not to have duly appreciated, owing to the monomaniacal focus on the decision problem at the heart of Behmann’s own views about the nature of logic.

Still, Behmann’s notes do not end with the dismissive assessment of Hertz’ work as “utterly trivial”, nor with outlining how Hertz’ goals can be reached by other methods, but in fact go on to engage at some length with another aspect of Hertz’ work, namely, the diagrams Hertz’ used to illustrate his abstract concepts and ideas.^62^ A sizeable portion of Behmann’s notes is devoted to such diagrams.

## Diagrammatic reasoning about (In-)Dependence in Hertz & Behmann

Some of the diagrams included in Behmann’s notes are taken straight from Hertz, other seem to be Behmann’s own invention. Like Hertz, Behmann presented some diagrams that visualise axiom systems for a given set of sentences involving finitely many elements (represented by nodes), where the axioms are explicitly represented by arrows, whilst the (derivable) sentences, which are not axioms, are left unrepresented. So, for example, the following two squares visualise two different axiom systems for the set comprising all sentences connecting four elements (the differences between Behmann’s squares and those in Hertz 1921, p. 155 and Hertz 1922, p. 248 are relatively minor: Hertz does not label the nodes but *does* represent also derivable sentences by dashed arrows, and Behmann rotated the second square 90° clockwise):

**Figure:**
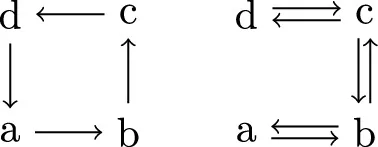

In the axiom system represented by the left square, for example, the sentences ‘a→b’, ‘b→c’, ‘c→d’ and ‘d→a’ are axioms, whilst, e.g. the sentences ‘b→a’ is derivable from the axioms ‘b→c’, ‘c→d’ and ‘d→a’ by transitivity of ‘→’. In the axiom system represented by the right square, there are six axioms (represented by the six arrows), so this axiom system, in contrast to the one with four axioms, is not minimal (as Hertz 1921, p. 155 points out).

But Behmann’s presentation goes well beyond merely reproducing the diagrams found in Hertz. Behmann also presents new kinds of diagrams geared towards purposes other than those Hertz’ figures served. For example, Behmann proposes to use diagrams to visually determine (anachronistically speaking) which truth-value assignments to elements would satisfy a given set of sentences (and thus to recognise, by means of a diagram, whether a system is consistent):

**Figure:**
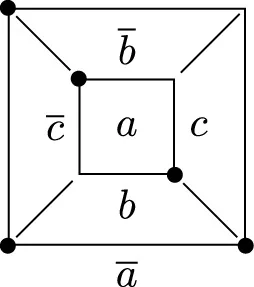

In this diagram, which Behmann calls simply: “Cube” and which has no parallel in Hertz’ work, the faces of the cube represent elements (with opposing faces representing each the negation of the opposite face: considering negated elements is another step Behmann takes beyond Hertz) and the vertices represent possible components of a conjunctive normal form (namely, each the disjunction of the element associated with the three faces which meet at the vertex), so, e.g. the top left corner in the picture (i.e. top, front, left vortex of the cube) represents the disjunction of ¬a (the *front* face), ¬c (the *left* face) and ¬b (the *top* face of the Cube). The corners marked by heavy dots finally represent a selection of such components, which spans a set of sentences; namely, those sentences which have conjunctive normal forms consisting of (some of) these disjunctions as components, see p. 42. So the Cube visualises the set of sentences having conjunctive normal forms with at least one of the following five components:The consistency of this system of sentences can be read off of the diagram—although Behmann does not explain this—by checking whether it is possible to, as it were, shade the faces of the cube in such a way that all selected corners adjoin at least one shaded faces whilst no opposing faces are shaded; in this particular case, shading the faces *b* and $\overline{c}$—i.e. (anachronistically speaking) assigning the truth-value true to *b* and the truth-value false to *c* (and whichever truth value to *a*)—will show the consistency of this set of sentences.

Behmann suggests furthermore that an axiom system for this set of sentences can be read off the diagram, and that it will be unique “if one requires that the axioms be split up as far as possible”, i.e. contain as few elements as possible (but at least 2, since Hertz’ sentences are always conditionals). Although Behmann simply states the following four axioms without explaining how exactly he derived them from his Cube-diagram, there is, upon reflection, a method underlying his choice: One chooses as axioms the disjunctions of two elements corresponding to the edges connecting selected vertices, so in this case:This choice will indeed be uniquely determined by the diagram, but will not—contrary to Hertz’ main ambition—yield a system of *independent* axioms: the above four are, in fact, still redundant (as I discuss below).

Finally, based on the same idea (foreign to Hertz) of representing sentences by way of the components of their conjunctive normal form, Behmann presents another kind of diagram not found in Hertz,^63^ which provides a visual test of sufficiency and independence:

**Figure:**
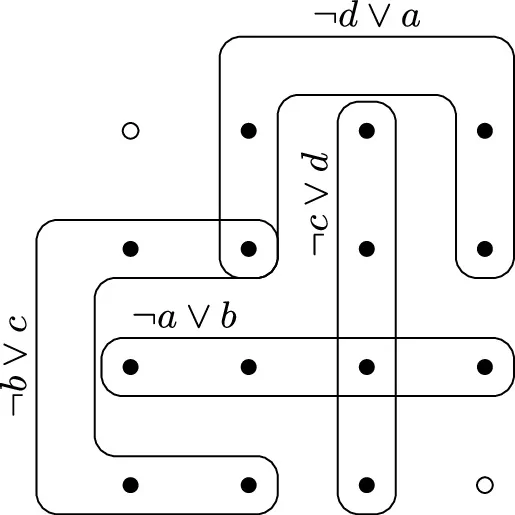

Behmann marks all the possible components of a conjunctive normal form (in the following diagram of four elements, see p. 44 for the corresponding table of components) on a grid, and fills in those that span a given set of sentences.^64^ Then Behmann visualises the, so to speak, “range” of the axioms by encircling all those selected components which a given axiom implies. In this diagram, the axioms are those illustrated by the first of the two squares discussed on p. 23, i.e. ‘a→b’, ‘b→c’, ‘c→d’ and ‘d→a’. Behmann considers, instead, the equivalent disjunctions: ‘$\lnot a\vee b$’, ‘$\lnot b\vee c$’, ‘$\lnot c\vee d$’ and ‘$\lnot d\vee a$’ (Behmann uses overline-notation for negation), and encircles for each all those nodes corresponding to such a component of a conjunctive normal form as contains the disjunction equivalent to the axiom as a part (which will be entailed by the axiom on account of $\phi \vee \psi \vdash (\phi \vee \psi )\vee \chi$). If all nodes are encircled, the system of axioms is adequate as such, that is to say: it entails all sentences in the given system (since for any component of a conjunctive normal form of such a sentence at least one axiom entails that component).

Furthermore, if none of the resulting closed curves lies entirely within (the fusion of) some other(s)—that is to say, all the nodes it comprises (the precise shape of the closed curve is immaterial) are also comprised by some other closed curve—the axioms are also independent because there is then for each at least one node that lies only in *this* axiom’s closed curve, thus (anachronistically speaking) at least one assignment of truth values to elements (namely the negation of the disjunction represented by that node) which will falsify this but no other axiom. So one can also read simple countermodels off the diagram.

Now, for an example where this test reveals the dependence of axioms, consider again the axiom system visualised by Behmann’s Cube discussed on the previous page. The axioms Behmann reads off of his cube are, in fact, dependent, as Behmann notes, though without drawing any diagram—a few attempts to combine three of these axioms to obtain the fourth will show as much—but one might also use a diagram like the above to ground this suspicion:

**Figure:**
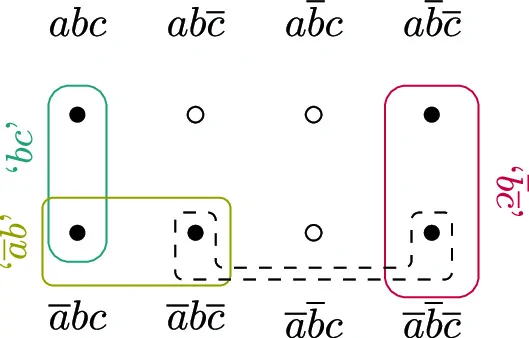

Here the (dashed) closed curve, which represents the axiom ‘$\lnot a\vee \lnot c$’, lies entirely within the fusion of the closed curves which represent the axioms  and . Consequently, ‘$\lnot a\vee c$’ is dependent and hence dispensable as an axiom (or, one could retain ‘$\lnot a\vee c$’ and drop ‘$\lnot a\vee b$’, whose corresponding closed curve lies entirely within the fusion of the curves corresponding to ‘b∨c’ and ‘$\lnot a\vee \lnot c$’).

To recognise that ‘$\lnot a\vee \lnot c$’ is indeed dependent, another ‘trick’ Behmann mentions in his presentation (see p. 43, line 5) comes in handy, namely, ‘read’ the disjunctions as conditionals (with the negated disjunct as the antecedent, or, if both are positive, the negation of either disjunct as antecedent). So the problem at hand is transformed into recognising that ‘$a\rightarrow \lnot c$’ follows—as the diagram suggests—from  and  (by the transitivity of ‘→’).

After dispensing with the dependent axiom ‘$\lnot a\vee \lnot c$’—the remaining axioms are independent as the following diagram shows—none of the closed curves representing the remaining three axioms coincides entirely with another:

**Figure:**
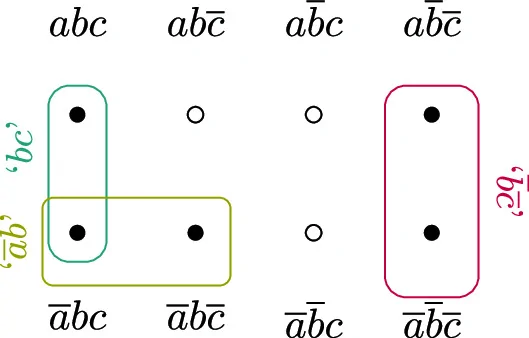

As I said before, one can read off the diagram assignments of truth values to the elements which establish the independence of the remaining axioms by considering the negation of any node that is comprised only in one of the closed curves. So, for example, the negation of the bottom, right node (‘$\overline{a}\overline{b}\overline{c}$’), i.e. the assignment $a\equiv \top$, $b\equiv \top$, $c\equiv \top$ satisfied‘b∨c’ & ‘$\lnot a\vee b$’, but not ‘$\lnot b\vee \lnot c$’; the negation of the top left node (‘*abc*’), i.e. the assignment $a\equiv \bot$, $b\equiv \bot$, $c\equiv \bot$ satisfies ‘$\lnot a\vee b$’ and ‘$\lnot b\vee \lnot c$’, but not ‘b∨c’ is not; and finally the negation of the second bottom node (‘$\overline{a}b\overline{c}$’), i.e. the assignment $a\equiv \top$, $b\equiv \bot$, $c\equiv \top$ satisfies ‘b∨c’ and ‘$\lnot b\vee \lnot c$’, but not ‘$\lnot a\vee b$’.

Apart from the interest that attaches to Behmann’s diagrams as examples of diagrammatic reasoning in (meta-)logic, highlighting the considerable thought Behmann put into developing Hertz’ ideas of using diagrams to visualise different aspects of dependence for his presentation on Hertz’ work is of some historical interest also because Behmann later employed “graph-theoretic considerations” for (meta-)logical purposes in his correspondence with Kaufmann & Gödel (as has been discussed at some length by: Mancosu 2010a, pp. 204 sq.).

In fact, something like the “star figures” Behmann used in that context can be found already in Hertz 1921, p. 156 sq. figs. 4b, 5a & 5b and again in Hertz 1922, p. 248 fig. 4b. Since Behmann familiarised himself with Hertz’ work intimately in 1922, and devoted special attention to Hertz’ diagrams, as attested by Behmann’s notes which include various Hertz-like diagrams, and also put them to uses not envisioned by Hertz, it seems to me that Behmann must have gotten “the idea of applying to proofs [...] graph-theoretic and topological considerations [...] [for] the first time” (Mancosu 2010a, p. 205) from Hertz.

## Hertz’ proof-theoretic project (II): results

In pursuit of his declared goal—formulating a criterion for the existence of a unique, independent axiom system yielding a given system of (what Hertz calls) sentences—Hertz arrives, finally, at the following (partial) result: Multiple distinct but all independent axiom systems for a given system of sentences exist only if at least two sentences in the system are non-trivially inter-deducible. This is a rough paraphrase of the theorem Hertz 1923a, p. 101 states in art. 94.^65^

To explain this result I will explain first how exactly Hertz conceptualises what I just called “non-trivial interdeducibility”, and then turn to what stands probably as Hertz’ most lasting contribution to proof theory, normal-form results for deductions, which serve in Hertz’ own work to make his abstract criterion for the existence of an independent axiom system applicable in practice.

### The concept of a cycle

In his own terms, Hertz’ theorem concerns what Hertz calls “cycles”, which Hertz 1923a, art. 89 defines as a “cyclically ordered system of sentences which are not tautological, not abundant and such that the succedent of each is contained in the antecedent of the previous one [according to the cyclical order]”.

**Figure:**
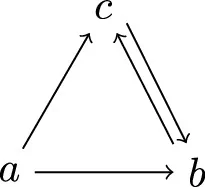

In its simplest form, such a constellation is illustrated by the square diagrams Hertz and Behmann present (see p. 23), or, in its minimal manifestation, by this diagram (taken from Behmann, who presumably models it on a similar triangle illustrating a cycle in Hertz 1921, p. 155 and Hertz 1922, p. 248). Here, two elements *b* and *c* are related by two sentences, ‘b→c’ and ‘c→b’, which constitute a cycle—however they are arranged in a sequence—because the antecedent of one is the succedent of the other. Tracing the relation of dependence amongst elements as expressed by these sentence would, starting from either *a* or *b*, eventually inevitably lead back to the same element, and never come to a stop, hence the name “cycle”.

Cycles arise from what Hertz calls “webs”, which are (according to the definition in art. 87, using the terminology introduced in art. 84 , see Hertz 1923a, p. 99) classes of mutually inter-deducible sentences, i.e. such that each member of it is deducible from any other in the web, and conversely the latter sentence from the former. “Web” is a more general concept, since webs, unlike cycles, need not be (linearly ordered) sequences; cycles present themselves as closed paths through webs, but one web can encompass many cycles consisting of very different sentences (though any two cycles in a web must overlap in at least one element). Since every web gives rise to at least one cycle (a result explicitly stated in Hertz 1923a, p. art.91), the presence of a cycle in a sentence system is a necessary condition for the presence of a web, and consequently the question if a cycle is present in a sentence system provides a criterion to determine whether there are non-trivially inter-deducible sentences in the given system, even if in many cases not only a cycle, but a whole web may be present. Cycles, as Hertz conceives of them, are the minimal indicators of non-trivial interdeducibility.

The restrictions Hertz imposes on members of cycles in his definition, that they must not be either “tautological” or “abundant”, accordingly serve to exclude different forms of redundancy and so ensure the minimality of cycles.

Hertz defines “tautological” (in art 7 of Hertz 1923a, p. 81) as meaning that a sentence has the same element both as antecedent and succedent; Hertz 1923a, art. 8 introduces also a more general term for sentences with multiple antecedents, which he calls “trivial” in case they have as succedent *one of* their antecedents. Any given cycle can be (redundantly) enlarged by adding tautological sentences. E.g. one could add the tautological sentence ‘b→b’ to the sequence ‘b→c’, ‘c→b’ to obtain ‘b→c’, ‘b→b’, ‘c→b’, which would also qualify as a cycle if it were not for the condition that excludes tautological sentences from cycles. In fact, ‘b→c’, ‘c→b’ already attests the interdeducibility of *b* and *c*, and a tautological sentence can only obscure this by rendering the would-be-cycle attesting interdeducibility needlessly convoluted.

On the other hand, tautological sentences can never create a cycle where none exists without them: For instance, the sequence ‘b→b’, ‘c→c’ does not constitute a cycle by itself; it becomes one only if ‘b→c’ and ‘c→b’ are added to the sequence—which, however, constitute a cycle by themselves.

Trivial sentences differ on this point: Consider, for example, the sentence ‘ac→a’ (which is trivial because its succedent, *a*, is *amongst* its antecedents). Together with the sentence ‘a→c’, this trivial sentence would constitute a cycle (if trivial sentences were admitted as members of cycles) even in case the sentence ‘c→a’ was not, itself, part of the underlying sentence system $\mathfrak {S}$ (as, e.g. in the system illustrated by the triangle on p. 27). In this case, *a* doesn’t actually depend on *c*. But *a* does depends on itself, and still does so also when *c* is present additionally. So the kind of sequence Hertz calls “cycles”, where each member has as a succedent (one of) the antecedents of the prior member, are reliable indicators of genuine deductive dependence only if trivial sentences are excluded, which they are thanks to the second condition Hertz imposes.^66^

In a setting where at least some sentences have multiple antecedents, a sentence is called “abundant” relative to an underlying system $\mathfrak {S}$ of sentence (according to Hertz’ definition in art. 57 of Hertz 1923a, p. 93) in case it differs from some other sentence also available in the system $\mathfrak {S}$ only by having more antecedents. Non-tautological trivial sentences are always abundant because they can be obtained by adding antecedents to the tautological sentence with the same (antecedent and) succedent, and within a closed system of sentences, all tautological sentences about elements occurring in any sentence of the system are always available (as Hertz stipulates in art. 24). So, taken together, the two conditions, excluding tautological and abundant sentences, also exclude trivial sentences (as one instance of abundant sentences).

There is, however, also another kind of redundancy precluded by the exclusion of abundant sentences from cycles: Recall the example of sentences connecting three elements *a*, *b*, *c* from p. 27: The sequence ‘ab→c’, ‘c→b’ also constitutes a cycle, but ‘ab→c’ is abundant because in the system presented on p. 27 the sentence ‘b→c’ is available, and ‘ab→c’ differs from ‘b→c’ only by the added antecedent ‘*a*’. As with the exclusion of tautological sentences, which cannot bring about interdeducibility, only make it harder to recognise by making cycles longer than they need to be, barring abundant sentences from cycles likewise ensures that cycles are recognised in their simplest form, i.e. at the level of sentences involving a minimal number of interdependent elements.

Whenever a system of sentences comprises a cycle of non-trivially inter-deducible elements, there is no *unique* minimal axiom system, as Hertz 1922, p. 248 observes and illustrates with these two diagrams, where the dependence amongst the three elements *a*, *b*, *c* is axiomatised in different but equally minimal ways: once by ‘a→b’, ‘b→c’, ‘c→a’, and once by ‘a→c’, ‘c→b’, ‘b→a’.

**Figure:**
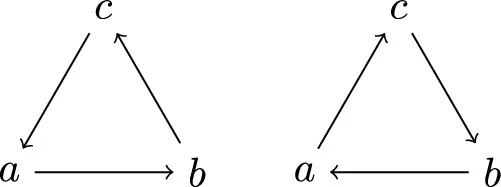

Whatever else the system of sentences might contain, apart from the inter-deducible elements connected by sentences constituting a cycle, the entire system will not posses a *unique* minimal system of axioms, because the cycle present in it can be so axiomatised in *different* but equally minimal ways.

In such cases, Hertz proposes considering what he calls Hertz 1922, art. 36 a “reduced” system of sentences, where a single element is picked as a representative of all the elements involved in the original cycle—or web through which the cycle at hand is a path (amongst others). Such a reduced system, where all webs and cycles present in the original system of sentences have been thus reduced to one element, representative of the position of the cycles/webs in the overarching dependence structure represented by the sentences in the system, contains no cycles (Hertz 1922, art. 38) and will consequently have a unique minimal axiom system (Hertz 1922, art. 41); and from it a minimal axiom system can be obtained also for the original system by adding one arbitrarily chosen minimal axiom systems for each cycle (cf.: Hertz 1922, art.42).

### Hertz’ normal-form result(s) for deductions

The concept of cycle is an abstract concept capturing a certain feature of the internal ‘structure’, so to speak, of a system $\mathfrak {S}$ of sentences, namely, whether somewhere in $\mathfrak {S}$ there are sentences expressing the *mutual* interdependence of certain elements on one another. In practice, the dependences obtaining between different elements which occur as constituent parts of the sentences comprised in a given system $\mathfrak {S}$ will not be surveyed all at a single glance, however, and so one is, in fact, rarely in the position of knowing whether there exists a cycle within a given system of sentences and so not equipped for applying Hertz’ criterion to decide whether the given system of sentences has multiple distinct, independent yet minimal axiom systems. “But”, Hertz says in the introduction of Hertz 1923a, p. 77, “we can avail ourselves of a convenient aid, which is that we can express the logical dependence amongst sentences in the form of a family tree”, i.e. using what Hertz calls “(primitive) inference systems” (see Sect. 3.3), and in this way figure out step by step which sentences the system contains, and which of them depend on which others (in a non-trivial way); for a more detailed discussion of the representation of deductions as trees in and before Hertz, see my forthcoming 2026.

Not every inference system whose conclusion is the same as one of its premisses reliably attests the existence of a cycle because the formulas used in the construction of the inference system may not, when arranged in a sequence, satisfy the defining conditions of cycles, since nothing about Hertz’ definition of a primitive inference system (see p. 15) precludes the use of trivial (in particular, tautological) or abundant sentences as lines.^67^

One might, therefore, consider only those specific primitive inference systems which happen not to contain either abundant or tautological sentences. Hertz calls such inference systems “inabundant inference system” and characterises them (in Hertz 1923a, p. 97) by means of the following three conditions:^68^ In addition to (a) containing no abundant sentence, an inabundant inference system also (b) must not contain any tautological sentences and (c) never uses the rule of inference Hertz calls “immediate inference”.^69^ Given the patent connexion between (a) and (b) and the defining conditions for cycles (see p. 27), the existence of such an inabundant inference system whose conclusion is the same sentence as one of its assumptions (from which it is deduced by more than one step) indicates the existence of a cycle, as Hertz 1923a, art. 93 claims.

Now all that remains is to ensure that these conditions are not overly restrictive: that, whenever there is a substantive deductive dependence amongst sentences in a given system, it can be recognised by means of an inabundant inference system in Hertz’ sense; i.e. that foregoing the use of abundant and tautological sentences as assumptions and intermediate steps, as well as the use of the rule Hertz calls “Immediate Inference”, does not constrain the deductive resources available too much, so as to obscure non-trivial dependences which obtain as a matter of fact but cannot be recognised by these specific means. This is where Hertz’ normal-form results come into play. Hertz establishes (in Hertz 1923a, art.73) that given a system $\mathfrak {S}$ of sentences any sentence which is not, itself, abundant (relative to $\mathfrak {S}$) or tautological, if it can be deduced from $\mathfrak {S}$ at all, can be deduced by means of an inabundant inference system.^70^

A crucial role in establishing this result is played, by way of a handful of intermediary results, by the theorem established in Hertz 1923a, art. 54. This theorem is Hertz’ main normal-form result, and, judging by the length at which Hertz argues for it, the culmination of Hertz (1923a).

In his argument to establish this result, Hertz goes through the modifications required in order to transform a given inference system into normal-form for every eventuality—for each inference rule—explicitly (like Gentzen later does in establishing his main normal-form result, Gentzen’s so-called “Hauptsatz”, for which the requisite modifications of given deductions are detailed in Gentzen 1935a, secs. 3.11f.). What Hertz establishes, is that any given inference system can be so modified as to give rise to a “normal proof”, which is an inference system where every major premiss (“Obersatz”, i.e. the premiss of Hertz’ syllogism/transitivity rule (see p. 15) whose succedent is the same as the conclusion’s) is what Hertz calls an “top sentence” (“oberster Satz”^71^), i.e. an assumption taken directly from the underlying system $\mathfrak {S}$ of sentences, rather than the conclusion of an earlier application of an inference rule.

Roughly but suggestively paraphrased, Hertz’ normal-form result, which is his Theorem 54, shows how to reconstruct a given deduction in such a way that every inference traces back the deductive dependence of one element, which appears as the succedent of the ultimate conclusion, *step by step* to more and more remote elements on which it depends. The relevant contrast is a deduction which proceeds through *compounding* deductive dependencies amongst these intermediary elements and shows only at the last step that the succedent of the ultimate conclusion actually depends on the the antecedent of ultimate conclusion by a single application a compounded dependence claim. (Hertz 1929b, §4 codifies the latter in its pure form by another normal-form result.)

An average deduction usually proceeds by a mixture of these modes.

Speaking less metaphorically, and more to the specific normal-form result(s) Hertz establishes: If information is available—in the form of the assumptions used in an inference system—that implies the deductive dependence of a conclusion *c* on $a_1,\dots , a_n$ (by way of intermediary elements $b_1,\dots b_k, d_1,\dots ,d_l,\dots$, which are dependent on the $a_i$s and on which *c* depends), then that information can either be ‘pooled’ into a single compounded dependence claim by (repeated) application of the transitivity of ‘→’ or it can be used piecemeal, one (in the given context) immediate dependence at a time, by (repeated) application of modus ponens. Since in Hertz framework there is only a transitivity rule (and correlatively no absolute conclusions or assumptions, see footnote 36), this difference manifests in whether the major or the minor premiss(es) serve as intermediary steps. Hertz 1929b, p. 473 provides the following two toy examples to illustrate the difference between these two normal forms of deduction:

**Figure:**
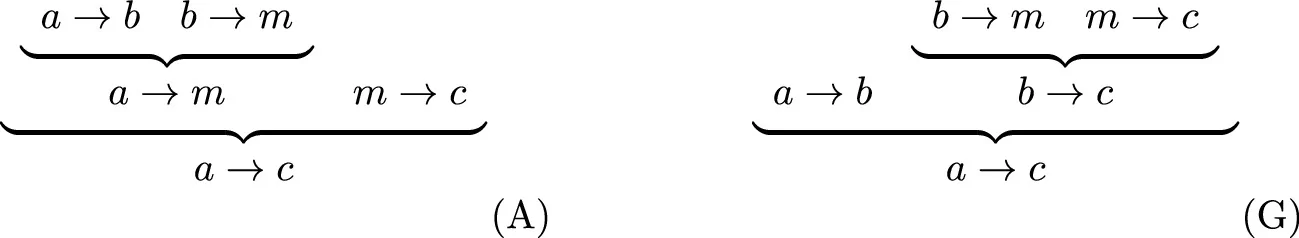

In the normal-form to the left (which Hertz calls “Aristotelian”), the minor premiss (‘a→m’, whose succedent *m* is *not* the ultimate conclusion *c*) is derived as an intermediary conclusion; in the normal-form to the right (which Hertz calls “Gocklenian”), by contrast, the major premiss (‘b→c’, whose succedent *c* *is* that of the ultimate conclusion) is deduced as an intermediary conclusion.^72^

Although exceedingly simple (e.g. in having only a single intermediary step), these examples also show that transforming a deduction—of, in both cases, ‘a→c’ from the assumptions ‘a→b’, ‘b→m’ & ‘m→c’—from one normal form into the other (or from any non-normal form into one of these two normal forms) generally requires a modification of the structure of the deduction, and in particular, changes are made to where in the deduction which assumption is deployed in support of which intermediary conclusion: In the deduction (A) ‘a→b’ and ‘b→m’ are used to deduce ‘a→m’ (which does not occur in (G) at all), whilst in the deduction (G) ‘b→m’ is used together with ‘m→c’ to deduce ‘b→c’ (which, in turn, does not occur in (A) at all).

### Normal proofs, cycles and minimal axiomatisations

It is not immediately obvious, given this characterisation of normal proofs (that every major premiss is an assumption), that such inference systems in the normal form provided by Hertz’ main result in art. 54 are, in fact, also such that they do not contain either abundant or tautological sentences.

Indeed, they do so only under certain further conditions, namely that the conclusion finally deduced is not itself abundant (or tautological), and that the sentences from the given system $\mathfrak {S}$ used as assumptions (what Hertz calls “top sentences”, see footnote 71) are also not themselves abundant.

These further conditions impose no real restrictions, however, when it comes to the search for cycles because, as I explained when discussing abundance (on p. 29): A sentence is abundant (by Hertz’ definition) if it differs from another sentence *available in the system* $\mathfrak {S}$ only by having some additional antecedents; but in case there is not merely an apparent cycle (as in the example ‘ac→a’, ‘a→c’ on p. 28), but a genuine cycle, then (as in the example ‘ab→c’, ‘c→b’ on p. 28) there must also be a cycle involving only inabundant sentences.

Although Hertz does not say so in so many words, he does presuppose from art. 60 onwards that the assumptions (top sentences) of the given inference system under consideration are taken not from a generic system $\mathfrak {S}$ of sentences, but from “a system [$\mathfrak {A}$] of inabundant axioms for a deductively closed system $\mathfrak {S}$ of sentences”. Hertz does not comment on this transition to explain why one can, without loss of generality, always presume the assumption of an inference system to be inabundant; or, rather, presume that for every inference system with a certain ultimate conclusion and assumptions from a given system $\mathfrak {S}$ of sentence, there is another (only slightly different) inference system with the same ultimate conclusion, all of whose assumptions, likewise taken from $\mathfrak {S}$, are inabundant. But a general result about abundance established in Hertz 1923a, art. 59 highlights the crucial fact that “any sentence which is abundant relative to a deductively closed system $\mathfrak {S}$ is strictly contained in an inabundant sentence”, i.e. deducible from an inabundant sentence by an application of immediate inference. So whenever one is considering an inference system with abundant sentences as assumptions, one can replace these assumptions with a single application of immediate inference whose premiss is an inabundant sentence. So a new, and only slightly different inference system can always be obtained, in which the same ultimate conclusion is deduced from inabundant assumption in the same way after the original, abundant assumptions have been deduced from the new inabundant ones by a single application of Immediate Inference. To the so modified inference system Hertz’ the procedure outlined in the proof for Hertz’ normal-form result can then be applied to excise the applications of immediate inference—which will, as I emphasised on p. 32, in general lead to a wide-ranging reconfiguration of the inference system.

Doing so will yield an inference system leading from inabundant assumptions to an inabundant conclusion of the general form (A), where all the major premisses are assumptions. If, now, such an inference system indicates circularity by having a major premiss like ‘$x\rightarrow a_i$’ (amongst) whose antecedent(s) is the succedent of the conclusion ‘y→x’, then the underlying system of sentences from which the assumptions used in this inference system are taken really does contains a genuine cycle, and so—by Hertz’ criterion—will not have a uniquely determined minimal axiom system. What is more, taking all the (non-tautological) major premisses starting from ‘$x\rightarrow a_i$’ down to the last major premiss in the order of their occurrence will, in fact, furnish precisely such a sequences of sentences from the underlying system that constitutes a cycle.

## Continuities between Hertz & Gentzen

Gentzen established in his Cut Elimination-“Hauptsatz”—which is like Hertz’ Theorem: a normal-form result for deductions in Gentzen’s (more sophisticated but basically similar) framework he called the “Sequent Calculus”—that any given deduction could be so modified as to not rely on the rule Gentzen called “Cut” (which is descendent from the rule Hertz called “Syllogism”, see footnote 45: also a transitivity rule for ‘→’).^73^ The avoidance of this rule is distinctive of an ‘especially perspicuous’ form of deduction in the sense that Gentzen regarded the Cut rule as signalling an argumentative “detour”, as Gentzen 1935a, p. 177 puts it: For deductions in which applications of the Cut rule are permitted may be such that in them concepts are used “which are not contained in the final result and thus [not] necessary for its derivation”.

Gentzen gloss on avoiding detours as a matter of avoiding concepts “not necessary for obtaining” the ultimate conclusion might remind the reader at this point of my description of Hertz’ motivation for establishing normal-form results for deductions: In order to discern genuine cycles amongst sentences of a given system, Hertz used normal-form results to excise from given deductions elements, occurring in the antecedents of abundant sentences, on which the succedent of the ultimate conclusion doesn’t *really* depend; (it merely seems so, due to the circuitous route the deduction takes). So Hertz’ normal-form result can be described like Gentzen describes his own, as showing that it is always possible to avoid bringing in elements not “necessary for [the conclusion’s] derivation”. (However, the far greater sophistication of Gentzen’s deductive framework imbues this shared idea with a totally different meaning, since what is and what isn’t “necessary” for the derivation of a given conclusion in a given deduction in the respectively relevant sense differs for Hertz and for Gentzen.)

In the course of his discussion, Gentzen furthermore draws from his normal-form results the same reassurance as I suggested (on p. 31) it provided to Hertz: to infer that restricting the use of the available means of inference—by dispensing with Immediate Inference, as Hertz does, or, in Gentzen’s case: Cut—in the deductive system at hand does not restrict which conclusions can be obtained, because—and this is what the normal-form results show—everything that is deducible at all is, in fact, deducible in normal-form, without these rules. So consistency (at least for sentential logic) is, according to Gentzen 1935b, p. 405, “a trivial consequence” of his normal-form result because a contradiction “is deducible [...] [only by means of] Cut, and hence not deducible at all”, since everything that is deducible is deducible without needing to rely on Cut.

There are also, apart from these shared ideas, fundamental differences between Gentzen and Hertz: Gentzen—like Hilbert, unlike Hertz—directs his efforts towards establishing the consistency of a certain deductive system. Still, Gentzen also uses his normal-form result to establish other non-deducibility results about formulas not related to the *inconsistency* of the deductive system, such as the law of excluded middle, concerning which Gentzen 1935b, pp. 407 sq. argues that it is not intuitionistically deducible because, not being what Gentzen calls a “base sequent” in intuitionistic logic (unlike in classical logic), it would have to be deduced, which it can be only by means of Cut, so not at all—since, in the light of Gentzen’s Cut Elimination-*Hauptsatz*, everything that is deducible is deducible without using Cut. Furthermore, Ketonen 1944, §§28, 30 later adapted Gentzen’s methods also for establishing independence.^74^

In 1936 Gentzen provided a proof-theoretic argument to establish the consistency of a theory of arithmetic with induction. He thus made a major contribution to the development of proof theory—and so also to the advancement of mathematics itself—along the trajectory set by Hilbert (cited on p. 12): by considering a more potent theory than had thereto been (successfully) treated by proof-theoretic means; (Hilbert’s initial collaborator(s), Ackermann (and von Neumann), succeeded only in applying proof-theoretic methods to establish the consistency of an axiom system of arithmetic *without induction*).

The particular normal-form result from Gentzen’s work on the proof theory of logic, his *Hauptsatz*, did not play a role in Gentzen’s first “consistency proof” for arithmetic (in 1936). (However, in Gentzen’s *second* “Widerspruchsfreiheitsbeweis” (published in 1938), Gentzen describes the reduction of induction as transforming applications of induction into “a series of “Cuts”” (Gentzen 1938, p. 27)—which can then be attacked with Cut elimination.) Still, *a normal-form result* for deductions, albeit a different one, does nonetheless play a crucial role also in 1936; Gentzen 1936, §14 calls it (in a Hilbertian vein) a “reduction”. In fact, this Reduction result is a far more sweeping normal-form result than Cut Elimination: It has the effect of substituting a single complicated inference, which Gentzen calls “Kettenschluß” (Gentzen 1936, p. 542, for (almost) all others (except universal generalisation and induction).^75^ Gentzen himself describing this rule explicitly in such terms, as “conceived so *broadly* that it comprises everything that is required [...] All *changes of structure* are special cases of “Kettenschluss”.” (Gentzen 1936, p. 543). The effect of his Reduction result, replacing all inference rules with applications of the Kettenschluss rule, Gentzen glosses as “as it were changing the original *successiveness* of inferences into a *juxtaposition*” (Gentzen 1936, 544).^76^ Whatever the import and merit of this metaphor, it makes clear that Gentzen saw this result for what it was: a total reconfiguration of the structure of a given deduction.

This result plays a *crucial* role in Gentzen’s argument in two respects: In the first instance because the use of the ‘one size fits all’-rule, Kettenschluss, is what enables Gentzen to reduce applications of the induction rule in such a way as to yield a, in a certain sense, less complex deduction of the same (numerical) end-formula. This is accomplished, roughly, by replacing an application of the induction rule, whose conclusion is subsequently used to justify a numerical formula about *n* by an application of Kettenschluss juxtaposing n-1 copies of the induction-step for numerals 2 through *n* (and the base-step).^77^ In the second instance, Gentzen’s sweeping “Kettenschluss normal-form” underlies Gentzen’s assignment of ordinals to deductions in 1936,^78^ in such a way to impose a well-ordering on them which, in turn, enables his use of transfinite induction.

## Conclusion

In the introduction I emphasised that, although both Hilbert and Hertz were guided in their way by Hilbert’s original motto “to treat proofs as mathematical objects”, they developed different brands of proof theory and treated proofs as objects of different kinds: Hilbert seems to have conceived of the objects of his theory primarily as something like ‘markers’ of deducibility (from certain assumptions), and developed proof theory accordingly as a theory of provability.

Hertz, by contrast, conceived of them as (non-linearly) ordered “inference systems” with a distinctive syntactic structure that can be manipulated and studied for its own sake. Hertz approach thus aligns with that programmatically outlined by Stanisław Zaremba (one of the few precursors explicitly cited in Hertz 1922, p. 247) in 1916. After noting that, so far, mathematical logicians—Zaremba names in particular “Peano and his students”—have so far spent more time devising notations for the constituent parts of deductions “than to studying deductions as wholes”, Zaremba 1916, p. 5 emphasises:

it is necessary to become acquainted, in their essential traits, with the structure and the properties of mathematical deductions.

Taking his cues from the sketch Hilbert presented at the international mathematical congress in Heidelberg in 1904, Paul Hertz’ proof-theoretic work offered a new perspective on the subject, which was to inspire much what subsequently—in the wake of Gentzen—came to be called “structural proof theory”.

The care and effort Hertz devotes to formulating, establishing and employing normal-form results for entire deductions testifies to Hertz’ interest in the structure of deductions. Hertz undertook this effort in service of the ultimate aims of his project: to determine whether a given system of sentences possesses a *uniquely determined* minimal axiomatisation (all in Hertz’ sense, see Sect. 3), for which purpose Hertz found he needed to discern under which conditions a given inference system revealed genuine (and in particular, genuinely circular) deductive dependencies amongst elements involved in it (see Sect. 6). But even merely introducing a conception of deductions, for which the precise (non-linear) order of steps matters, as it does for what Hertz calls “inference systems” (discussed in Sect. 3.3), is another indication of Hertz’ interest in structural features of deductions, seemingly at least to some extent simply for the sake of it.

That Hertz regarded the structure of deductions as intrinsically interesting is attested also by the following result, which Hertz 1923a, art. 56 derives from his normal-form result (in art. 54) as an immediate corollary, seemingly for its own sake (seeing how he never uses it subsequently:^79^

If e is the lowest sentence in a primitive normal inference system, and $\mathfrak {e}'$ a sentence which is not an uppermost and not the lowest sentence of the system, then the antecedent of $\mathfrak {e}'$ is contained in the antecedent of $\mathfrak {e}$.

Looking back at the toy example (A) on p. 32, bears this out: The lowest sentence, i.e. the ultimate conclusion, is ‘a→c’, its antecedent is ‘*a*’. The only sentence in the inference system which is neither an uppermost sentence (i.e. an assumption), nor the conclusion, is ‘a→m’, and, indeed, its antecedent, ‘*a*’, is contained (indeed, identical with) that of the ultimate conclusion. The more complicated example of an inference system in Aristotelian normal form Hertz exhibits on Hertz 1923a, p. 80 corroborates this general result as well. Gentzen’s concept of an “endpiece” of a deduction (for which, see: Gentzen 1938 pp. 28 sq.) codifies similar structural information

Making allowance for the extreme simplicity of Hertz’ framework, one can even recognise in it something like a precursor to what Prawitz 2006, §III.2, Cor. 1 later called the “subformula principle” with respect to normal deductions in natural deduction calculi.

So, the importance of Paul Hertz’ work for the development of structural proof theory can hardly be overestimated,^80^ seeing how Hertz pioneered ideas and results that came to be amongst the characteristics of structural proof theory: normal-form results for deduction (see Sect. 6), established by arguments by induction on the construction of proofs (see footnote 73); specifically, by considering for each inference rule how to transform applications of it (in certain contexts, see the proof of Hertz’ normal-form result in Hertz 1923a pp.87–93). Last but not least, I have highlighted what seems to me one crucial, so to speak, “epistemic” gain normal-form results in proof theory afford: They ensure that restricting the intension of provability—by disallowing the application of certain rules—does not correlatively restrict the extension: everything that is provable at all can—as normal-form results for deductions mainly show—be proved in some especially perspicuous form.

Another case, where ground-breaking advances in the evolution of proof theory coincided with the formulation and/or application of a normal-form result for the ‘syntactic’ objects central to proof-theoretic considerations, is provided by the normal-form results for complex terms and formulas, which proved a crucial tool to Hilbert when he set out in 1920–1924 to establish the consistency of theories containing enough arithmetical substance—such as at least the basic modes of calculation—for the assorted consistency results to hold any serious mathematical interest.^81^ Normal-form results for entire deductions were an integral part for Hertz’ brand of proof theory, and played a similar role in Gentzen’s work, which, though it pursued Hilbert’s original goals (of establishing the consistency of (ultimately) classical analysis), did so by means of methods inspired primarily by those pioneered by Hertz

## Data Availability

No quantitative data are used.

## Appendix: Heinrich Behmann’s notes on Hertz

As noted in footnote 59, the short manuscript transcribed and translated here is found amongst Behmann’s papers, now held by the Staatsbibliothek Preußischer Kulturbesitz in Berlin (SBB) as Nachlass 335, in Box 9 as Item 33.

**Table:**

Heinrich Behmann.	
*Zum Problem der Axiomatik vom Standpunkt der Algebra der Logik*	
[*Concerning the Problem of Axiomatics from the Standpoint of the Algebra of Logic*]	
28. Nov. [19]22.	
*Bemerkungen* zu dem Vortrag von H[ertz]. *Neues und bemerkenswertes Problem* aufgeworfen. Allerdings noch recht *speziell*. a→b, ab→c. *Allgemeinere Grundlage*, geeignete für die Behandlung. Alles sehr naheligend! Kein tieferer Gedanke nötig. Eine *Vorbemerkung*.	Remarks about the presentation of H[ertz]. Raised new and noteworthy problem. But everything still rather *specific*. $a\rightarrow b, ab\rightarrow c$. *More general foundation*, suited for treatment. Everything very obvious! No deeper thought needed. A preliminary remark
Eine *Vorbemerkung*.	A preliminary remark
I. $$R,a,b,c\dots f \left\{ \begin{array}{c}(x).xRx,~(x,y,z):xRy.yRz.\supset xRz\\ aRb,~cRf,~dRa~\text {u.s.w.}\end{array}\right. $$	
Schon specielles höheres Problem.	Already a specific higher problem.
II. *Implikation*. Also *Deutung* von *R*. Dann im Gegenteil *trivial*. (Grundsätzlich betrachtet!) Aber in gewissem Sinne der *natürlichste Ausgangspunkt* für das allgemeinste Problem. (Z. B. *Geometrie*!).	II. *Implication*. That is *interpretation* of *R*. Then, to the contrary, *trivial*. (Considered in principle!) But in a certain sense *most natural point of departure* for the most general problem. (E.g. *geometry*!).
*Zu II* eine sehr naheliegende *Verallgemeinerung*.	A very obvious *generalisation* of II.
Wer mit *Logikkalkül* näher *vertraut* ist, [wird]^1^ auffallend finden, daß, obwohl die Sachverhalte sich als einfach und durchsichtig ergeben, doch *tiefe und verwickelte Überlegungen* notwendig erscheinen, um zu ihnen^2^ zu gelangen, obwohl das Problem sich ja noch ganz innerhalb der *untersten Logikstufe* bewegt, wo ja alles vollkommen *trivial* geworden ist.	Whoever is *familiar* with the *logical calculus*, [will] find striking that, though the facts seem easy and transparent, still *deep and tangled considerations* seem necessary to get to them, though the problem still moves on the *lowest level of logic*, where everything has become totally *trivial*.

^1^ The sentence is lacking a main verb in the manuscript. I have supplied one.

^2^ Before these words, the word “sie” has been written, but struck out.

**Table:**

Namentlich da das *Entscheidungsproblem* auf diesem Gebiet bereits *seit Langem gelöst* ist, und alle Zusammenhänge, vor allem^3^ auch der *Begriff des Beweises* von außerordentlicher Trivialität geworden.—Natürlich *nicht* Beweisbarkeit logischer Formeln *aus den Axiomen der Logik*! Darum handelt es sich hier nicht, sondern nur etwas viel Einfacheres. Die Logik ist sich hier selbst bereits voraus! (Das Rein Logische versteht sich immer von selbst.)	Because the *decision problem* in this realm is *long since solved*, and all connexions, and also the *notion of proof* has become of an exceptional triviality.—Of course, *not* provability of logical formulas *from the axioms of logic*! That is not what is at issue here, rather something much simpler. Logic is here already ahead of itself! (Whatever is Purely Logical always is of itself intelligible.)
Dabei das *schwere Rüstzeug der allg. Beweistheorie* bei der allgemeinsten Problemen auf dieser Stufe noch gar nicht angebracht. *Nicht*: *beliebiger* Beweis, | umformen in Normalbeweis, sodaß er gewissen Bedingungen genügt; viel einfacher: *ein bestimter* Beweis, durch das Entscheidungsverfahren gegeben^4^.	For this the *heavy machinery of gen[eral] proof theory* is not appropriate even for the most general problems on this level. *Not*: *arbitrary* proof, transform into normal proof, such that it satisfies certain conditions; much easier: *a certain* proof, supplied by the decision procedure.
Allerdings zugleich *Verschiebung in das Interesse* an den einzelnen Fragestellungen, die zu einem großen Teil an der speciellen Form a→b und ab→c hängen. Aber trotzdem geeignete Grundlage.	However, at the same time *shift in the interest* in the particular questions posed, which for the most part attach to the specific form a→b and ab→c. But still a suitable foundation.
*Problem*. a,b,c⋯f *feste Aussagen*. *Inhalte* kennen wir^5^ nicht, nicht einmal *Aussagenwert*. Aus einem *beliebigen Wissensgebiet*. *Nicht* aus der *Logik*; diese setzen wir voraus.	*Problem*. a,b,c,⋯f *fixed statements*. *Contents* not known to us, not even *statement-value*. From an *arbitrary realm of knowledge*. *Not* from *logic*; this we presuppose.

^3^ Before these words, the word “nament[lich]” has been written, but struck out

^4^ Kneser’s student notes for the lecture of 23/XI/1922 record Bernays making a similar remark, namely that “the “coming [from an assumption to a conclusion]” works for the small sentential calculus because there universal truth can be read off the normal-form. To effect this in the case of the “larger” [functional] calculus is the *Decision Problem*.” (Hilbert 2013, p. 608).

^5^ Before these words, the same words have been written once already, but struck out.

**Table:**

Wir kennen nur *bestimmte Zusammenhänge* zwischen den Grundaussagen, Aussagen über sie, die richtig sein sollen^6^.^7^Aussagenoperationen. Verknüpfung nur durch (nicht, oder, und, folgt, äquivalent).	We know only *certain connexions* between the basic statements, statements about them, which are supposed to be correct. [These are called *axioms*.] Operations on statements. Connexions only by means of (not, or, and, implies, equivalent to).
Einen Teil der Aussagen, aus dem alle anderen folgen, nennen wir *Axiome*. *Überblick über alle möglichen Axiomensysteme*!^8^	Some of the statements, from which all others follow, we call *axioms*. *Overview of all possible axiom systems*!
*Axiom*. Begreift noch Willkür. Zerspaltung. A[NONSPACE]&B.	*Axiom*. Still contains arbitrariness. Splitting. A[NONSPACE]&B.
Man braucht also nur *Disjunktionen*. *ab*, $\overline{a}b$, $a\overline{b}\overline{c}$ u.s.w.	Thus one needs only *disjunction*. $ab,\overline{a}b,a\overline{b}\overline{c}$ a.s.o.
(Erinnerung an Normalform!)	(Recall normal-form!)
Darin stecken bereits a→b und ab→c. *Ausgangsfall* also insofern *allgemeiner* als H. Dort mindestens ein Glied überstrichen und *genau* ein Glied unüberstrichen.	In this a→b and ab→c are already contained. *Initial case* insofar *more general* than H[ertz]. There at least one member overlined and *exactly* one member not overlined.
*Scheinbar spezieller*, aber nur für den *Augenschein*. Bei H. *formale* = *allgemeine* Implikationen. Dies aber *gar kein Unterschied*. Alle^9^ Betrachtungen sind genau dieselben. Man kann statt *a*, *b*, *c* *f(x), g(x), h(x)* geschrieben und vor jede hingeschriebene Aussage noch *(x)* davor gesetzt denken. Bequemer, Variable zu *unterdrücken*.	*Seemingly more specific*, but only *at first glance*. With H[ertz] *formal* = *general* implications. But this is *no difference at all*. All considerations are exactly the same. One can say *f*(*x*), *g*(*x*), *h*(*x*) instead of *a*, *b*, *c* and preface each expression written down with (*x*)^11^. More convenient to *suppress* the variable.
*Aussagen lassen sich im allgemeinen noch zerspalten*. Sogar $\overline{a}b\leftrightarrow$ $\overline{a}b(c\cdot \overline{c})$^10^$\leftrightarrow \overline{a}bc\cdot ab\overline{c}$. |	*Statements can generally be split up further*. Even $\overline{a}b\leftrightarrow \overline{a}b(c\cdot \overline{c})\leftrightarrow \overline{a}bc\cdot ab\overline{c}$.

^6^ The manuscript originally had “als richtig gegeben we[rden]”, but “als” has been struck out and so has “gegeben we”, which has been replaced above the line with “sein sollen”.

^7^ Here the sentence “Diese heißen *Axiome*.” has been written, but struck out.

^8^ This sentence has been added above the line with an insertion mark

^9^ Before this word “Mei[ne]” has been written, but struck out.

^10^ Before this formula, “$\overline{a}bc\cdot \overline{a}b\overline{c}$” has been written but struck out

^11^ I.e. take its universal closure; ‘(*x*)’ being Behmann’s notation for the universal quantifier binding the variable ‘*x*’.

**Table:**

Wir fragen nach den *schwächsten* Aussagen, die überhaupt möglich sind.	We inquire about the *weakest* statements, which are at all possible.
$\overline{a}b\dots \overline{f}$. Wo alle Buchstaben verbraucht sind. Jeder genau einmal. Unüberstrichen oder überstrichen. $2^n$ Uraussagen, (= Atome)	$\overline{a}b\dots \overline{f}$. Where all letters are used. Each exactly once. Unoverlined or overlined. $2^n$ elementary statements (= atoms)
*Beispiel*. n=3.	*Example*. n=3.
$$abc, ab\overline{c}, a\overline{b}c, a\overline{b}\overline{c}, \overline{a}bc,\overline{a}b\overline{c},\overline{a}\overline{b}c,\overline{a}\overline{b}\overline{c}$$.	
*Jede Aussage* darstellbar durch Konjunktion von Uraussagen. Vollständig eindeutig. (Normalform war noch nicht eindeutig.) *Vollentwicklung*.	*Every statement* representable as conjunction of elementary statements. Completely unique. (Normal form was not yet unique.) *Full development.*
Wie steht es mit der *Wahrheit der Uraussagen*? *a*, *b*, *c* bestimmte Werte. Alle richtig mit genau einer Ausnahme. Also *unabhängig* von einander!	How about the *truth of elementary statements*? *a*, *b*, *c* fixed values. All correct with one exception. So *independent* of one another!
Was heißt: *ein Satz folgt logisch aus einem anderen* oder mehreren anderen. Die Voraussetzung erfüllt ein Atom, ebenso den Schlußsatz. *Enthaltensein*. Man braucht nur *zuzusehen*, ob alle Atome vorkommen.	What is the meaning of: *a sentence follows logically from another* or several others. The condition satisfies an atom, the conclusion as well. *Containment*. Merely *look* whether all atoms occur.
Was heißt: Ein *System von Sätzen ist widerspruchsfrei*? Wenn es nicht alle Uraussagen enthält (bei Zerspaltung). Die einfachste Uraussage bleibt dann außerhalb. *Widerspruch* dann und nur dann, wenn *alle* Atome vorkommen.	What is the meaning of: A *system of sentences is consistent*? If it does not contain all base statements (upon splitting). The simplest base statement remains outside then. *Contradiction* if and only if *all* atoms occur.
Entsprechend für *Axiomensystem*. *Widerspruchsfrei*, *Unabhängig*!$^{12}$ *vollständig*, falls genau n-1 Uraxiome^13,14^. Dann liegen alle Aussagenwerte fest.	Accordingly for *axiom systems*. *Consistency*, *independence!*[,] *complete*, if exactly n-1 elementary axioms. Then all values of statements are fixed.

^12^This word has been added above the line with an insertion mark.

^13^ The manuscript originally had “Uratome”, which has been struck out and replaced on the line behind the original formulation.

^14^ This is in line with Hilbert’s conception of completeness at the time, namely, that a system is complete if adding even one further statement to it would result in an inconsistent system. Since Behmann has just asserted that inconsistency is a matter of containing all all basic statements, a complete system will be any that (is correct and) contains all *except* one basic statement.

^15^The manuscript originally had “ein und nu[r ein]”, which has been struck out and replaced on the line behind the original formulation.

**Table:**

In einem gewissen Sinn giebt es stets nur ein einziges Axiomensystem,^15^ wenn man verlangt, daß die Axiome so weit wie möglich gespalten werden.	In a certain sense there is *always only one possible axiom system*, if one requires that the axioms are split up as far as possible.
	*Example*
	*Cube*.

	*axiom system* e.g. $bc, \underbrace{\overline{a}b,\overline{a}\overline{c}}, \overline{b}\overline{c}$ Not independent! Transformation $c/\overline{c}$ Then *implication* $\underbrace{b\overline{c},\underbrace{\overline{a}b,\overline{a}c},\overline{b}c}$
|	
*Erfüllungen*. Durch welche Aussagenwerte *a*, *b*, *c* wird ein gegebenes Axiomensystem erfüllt? Sehr einfach. Genau^16^ eine der nicht verwendeten Aussagen sei die^17^ *falsche*! Also in meinem Falle.	*Satisfaction*. Which values of statements *a*, *b*, *c* satisfy a given axiom system? Very easy. Let exactly one of the statements not used by the *false* one! So in my case.
$$\left. \begin{array}{c l c} \overline{ab\overline{c}} & \text {, d.h.} & \overline{a}.\overline{b}.c\\ \overline{a\overline{b}c} & \text {,} & \overline{a}.b.\overline{c}\\ \overline{\overline{a}\overline{b}c}& \text {,}& a.b.\overline{c} \end{array}\right\} $$ auf eine und nur auf eine Weise erfüllbar.^18^	$$\left. \begin{array}{c l c} \overline{ab\overline{c}} & \text {, d.h.} & \overline{a}.\overline{b}.c\\ \overline{a\overline{b}c} & \text {,} & \overline{a}.b.\overline{c}\\ \overline{\overline{a}\overline{b}c}& \text {,}& a.b.\overline{c} \end{array}\right\} $$ satisfiable in one and only one way.
Die *Erfüllungen* sind die *stärksten* Aussagen, die möglich sind. Jede Aussage läßt sich $^\dagger$mithin$^\dagger$ auch als Disjunktion^19^ von stärksten Aussagen schreiben. Zu der früheren Form dual. = *Auflösung des Axiomensystems*.	The *satisfactions* are the *strongest* statements possible. Every statement admits perhaps also of being written as the disjunction of strongest statements. Dual to the earlier form = *Resolution of the axiom system*.

**Table:**

Ist das Axiomensystem *vollständig*, dann *genau eine* Auflösung = Erfüllung.	Is the axiom system *complete*, then *exactly one* resolution = satisfaction.

^16^ Before this word “Ein” has been written, but struck out.

^17^ This word has been added above the line.

^18^Here the sentence “Eine der nicht verwendeten” has been written, but struck out.

^19^ Before this word, the indefinite article “eine” has been struck out.

**Table:**

	*Beispiel von H[ertz].*
	*Example from H[ertz]*$^{20}$

$\overline{a}b, \overline{b}c,\overline{c}d,\overline{d}a$ // $\overline{a}b,\overline{b}c,\overline{c}d,\overline{d}c,\overline{c}b,\overline{b}a$	
$$\begin{array}{c c c c} abcd & abc\overline{d}& ab\overline{c}d& ab\overline{c}\overline{d}\\ a\overline{b}cd& a\overline{b}c\overline{d}& a\overline{b}\overline{c}d& a\overline{b}\overline{c}\overline{d}\\ \overline{a}bcd& \overline{a}bc\overline{d}& \overline{a}b\overline{c}d& \overline{a}b\overline{c}\overline{d}\\ \overline{a}\overline{b}cd& \overline{a}\overline{b}c\overline{d}& \overline{a}\overline{b}\overline{c}d& \overline{a}\overline{b}\overline{c}\overline{d} \end{array}$$	

Beide Axiomensysteme sind *unabhängig*^21^	Both axiom systems are *independent*
Unterschied von *geometrischer* und *vollständiger* *Unabhängigkeit*. Parallelaxiom!	Difference between *geometric* and *complete independence*. Axiom of parallels.
*Keine* *gemeinsame* *Folgerung*!	*No common consequence*!
*Verallgemeinerung für höhere Aussagebereiche*? Durchaus möglich. Nach den gleichen Ideen, *soweit Entscheidungsproblem gelöst*. Damit auch alle anderen Probleme trivial geworden. $(x),(E\;x), (P), (E\;P),(x!)$ *Keine Deutungen mit höheren Klassen*!	*Generalisation for higher domains of statements*? Certainly possible. Using the same ideas *insofar as decision problem solved*. With that also all other problems trivialised. $(x), (E\,x),(P),(E\,P),(x!)$ *No Interpretation using higher classes*!

^20^The example is taken from Hertz 1921, p. 155.

^21^ “unabhg” has been written already at the end of the previous line, but struck out, presumably because the line was too short.

## Footnotes

1 As far as I am aware, Hilbert uses the word “Beweistheorie” for the first time in lectures in 1920 (see: Hilbert 2013, p. 367); for the first time in print in Hilbert 1935d p. 169. Text quoted only as supporting evidence in footnotes is quoted in its original language, whilst text quoted as part of the main text appears in English translation; these translations are my own throughout.

2 Cf. the emblematic description of these different approaches in Dieudonné 1974 sec. IV.4.

3 Kreisel 1984, p. 207 glosses consistency as “the weakest non-derivability result, since it is the problem of showing that there exists at least one statement which is not derivable”.

4 A similar description was offered by Prawitz (1974) p. 63: “Hilbert did not really create a theory, however; rather he introduced a certain research project which he named proof theory”.

5 This is the proof-theoreticians’ basic creed, ““irrefutable” by the methodological decision of its proponents” (Lakatos 1978, p. 50), it is what “characterises” proof theory as a research programme, its “hard core” or “negative heuristic”, as Lakatos 1978 p. 48 calls it.

6 For a brief overview of Hertz’ career, see section IV.15 in Kern 2011, pp. 112 sqq. (of the original pagination = pp. 46f. of the online edition). Hertz’ life and work is also concisely described by Pinl 1971. 173–176, which includes a comprehensive bibliography, and, even more concisely, in an entry in the *Neue Deutsche Biographie*, vol 8 (1969), pp. 711f. by Bernays. For a compact overview of Hertz’ work in proof theory, see Abrusci 1983.

7 For the former epithet, see: Einstein 1994, p. 314 (orig.p.175). The latter is quoted by Hertz’ son Rudolf from a condolence letter his mother received from Einstein when Paul Hertz died in 1940 on p. 11 of the biographical sketch kept with Paul Hertz’ papers at the Archive for Scientific Philosophy in Pittsburgh, and freely available online at: https://digital.library.pitt.edu/islandora/object/pitt%3A31735067430599/ (checked 3/IV/2026); the volume covering Einstein’s correspondence in 1940 has not yet been published.

8 In a cv submitted for his appointment as lecturer in Göttingen, which is found in Hertz’ personnel file kept by the Universitätsarchiv of the university of Göttingen, Hertz states the title of his dissertation as “Untersuchungen über unstetige Bewegungen eines Elektrons” , and the date of his obtaining the doctorate as July 28 1904.

9 Hertz’ personnel file reports the conferral of the title “Professor” by decree of the ministry of education on 7/III/1918 and of “Extraordinary Professor” on 7/XI/1921. The former title of “Professor” was a mere honorific conferred “in Anerkennung seiner wissenschaftliche Leistungen” (as the letter from the ministry dated 18/III/1918 puts it); it did not amount to an actual position at the university: Hertz remained an external lecturer (Privatdozent). The latter title of “Extraordinary Professor”, by contrast, was a “Dienstbezeichnung” (as the letter from the ministry dated 7/XI/1921 puts it), i.e. amounted to the conferral of a permanent position at the university (and was, as a letter from the ministry dated 3/IV/1922 attests) also soon (though not automatically) accompanied by a stipend, though not a salary.

10 As the correspondence between Hertz and Lorentz shows, especially the letter found amongst Hertz papers at Archive for Scientific Philosophy in Pittsburgh under the signature “PH 01-16-08” (dated 3/IV/1911), in which Lorentz asks what Hertz intends to work on during his stay in Leiden, so he, Lorentz, can prepare himself accordingly. (The letters found in Pittsburgh are all photocopies from Lorentz’ papers; I have not seen the originals.)

11 Hertz reports only the general topic of this work, but no specific title in his cv, saying that he obtained his Habilitation “mit einer Arbeit über die Theorie des Saitengalvanometers”.

12 This is the only one of Hertz’ articles to have been translated into English (by Javier Legris in 2012 for the collection Béziau 2012, pp. 11–29). A thorough summary and systematic discussion of its results (also in English) was more recently provided by Arndt 2019, sec. 3. Moriconi 2015, §§  2–4 and Franks 2010, pp. 366–369 also discuss the article (in less detail).

13 Whilst not available in translation, this article, too, is summarised in English, if only comparatively briefly, by Arndt 2019, sec. 7.

14 Published in the *Jahresbericht der Deutschen Mathematiker-Vereinigung* (vol. 30, 1921, p. 46 of the “Mitteilungen und Nachrichten”).

15 Sieg 2013a, p. 124 quotes from a letter of Bernays reporting on the meeting to Hilbert.

16 According to a report in the *Jahresberichte der Deutschen Mathematiker-Vereinigung* (vol. 33, 1924) Hertz presented to the Mathematische Gesellschaft in Göttingen on “eine Fortsetzung der in den Math. Annalen, Bd. 87 u. 89 erschienen Arbeiten “über Axiomensysteme für beliebige Satzsysteme.”” on 20/V/1924 (see p. 120 of the “Mitteilungen und Nachrichten”-section). The report goes on to specify the content of Hertz’ presentation as follows: “Auf die dort behandelten Fälle werden jetzt gewisse Fälle zurückgeführt, in denen die zu betrachtenden Sätze Variable [sic] enthalten.”, which sounds like a summary of Hertz’ work in 1929 on what he calls “Makrosätze” and “Mikrosätze”; in particular, the reduction here alluded to is a central step in Hertz’ later article (see: Hertz 1929b, p. 500)

17 As a report of a presentation on the “Kausalprinzip” on 15/VI/1926 (in the *Jahresberichte der Deutschen Mathematiker-Vereinigung* (vol. 35, 1926), p. 126 of the “Nachrichten und Mitteilungen”) indicates. An article by Hertz on causality entitled “über den Kausalbegriff im Makroskopischen, besonders in der klassischen Physik” appeared subsequently in the first volume of the newly founded journal *Erkenntnis* in 1930, pp. 211–227.

18 This was the designation of a salaried lectureship position conferred on Hertz by the ministry of education on 14/VIII/1922, as Hertz’ personnel file records.

19 Particulars concerning Hertz’ remuneration are reported in the personnel file extensively, e.g. its regular increases to keep up with the rampant post-WWI inflation in Germany. A vivid first-hand account of the material difficulty these years presented even to vastly more successful and well-positioned (secular Jewish) academics than Paul Hertz ever managed to become is found in the memoirs of Ernst Cassirer’s wife, Toni, see Cassirer 2003, pp. 146-149.

20 As Max Born reports in a letter found in Hertz’ personnel file (dated 16/VII/1922), meant to draw attention to Hertz’ “economic distress”: “Prof. Hertz and his wife have tried to subsidise their income by translation- and typewriting-work”. (For example, Hertz translated Russell’s *The Problems of Philosopy* into German in 1926.)

21 Particulars about Hertz’ salary and situation are reported in a letter by Hans Driesch to Moritz Schlick, dated 18/VI/1925, who also reports Hertz’ desire in 1925 to leave Göttingen; the situation seems to have remained unchanged, however, as Max Born reports to Schlick in a letter dated 16/IV/1931 that Hertz is still in Göttingen, still living on very little income.

22 This was noted by Max Born in a letter written in support for the application for the salaried lecturer-position mentioned in footnote 18. Born’s letter is also found in Hertz’ personnel file. In it Born describes Hertz’ work as follows: “However, [Hertz’] talents are mainly directed towards the critical, logical. So he has not taken part in any essential way in the rapid development of physical ideas taking place in recent years and has therefore fallen into a certain external disregard. This prompted him to turn to more abstract directions of thinking, and now he works in the border regions of philosophy, mathematics and physics.” (Compare Einstein’s recommendation letters quoted on p. 3, which likewise stress Hertz’ “critical talent”, though Einstein there also speaks of Hertz’ “remarkable originality”.)

23 Carnap records in his diary (Carnap 2022, p. 721, in the entry for 9/XI/1935) that Hertz complained to him about Reichenbach and James Franck sabotaging his chances of obtaining a position abroad by speaking unfavourably of him, though Carnap does not record what Hertz thought Reichenbach & Franck had said, nor to whom in particular; but Carnap does goes on to record: “both [Hertz and his wife] are very agitated and Mrs. Hertz is crying”.

24 In a letter to Moritz Schlick dated 12/IX/1920, Hertz confesses that whilst he would want most of all to devote himself mainly to philosophy, he had no hopes of making a career out of philosophy because of his lack of published philosophical work, as well as the nature of his work which he saw as lying outside the mainstream of philosophical research at the time.

25 So his son Rudolf reports in his biographical sketch (referred to in footnote 7, p. 11). Max Born likewise reports to Schlick (in a letter dated 16/IV/1931) that Hertz’ lectures on physics in Göttingen attracted a large number of students, who held them in high regard.

26 Einstein’s reference letters (quoted on p. 3) were part of an effort to bolster Hertz’ livelihood, since, as Einstein reports to Goldschmidt, Hertz was “infolge des Krieges in eine schwierige materielle Situation geraten”; in a letter *to* Einstein, Max Born described the situation more drastically (30/IV/1922, Einstein 2013, p. 290): “Ich habe an Paul Hertz, der ebenfalls [like Imre Brody, the main object of concern in this letter] am Hungertuch nagt, schon genug Sorgen und Verantwortung, für die ich nichts kann.”. Hertz’ son mentions this financial hardship on p. 9 of his biographical sketch (referred to in footnote 7).

27 Hertz describes himself in this way in a letter to Schlick dated 20/IV/1920. In a later letter to Schlick, dated 3/X/1926, Hertz writes that he has learned about himself that whenever he sat down to write even just an overview of something, these texts regularly “so anschwellen, daß sie schließlich ausführliche Ausführungen werden”. Hertz also regularly lamented the fact that he could not finish any of his projects in letters to Bernays (dated 22/V/1934 (ETH, HA Hs 975:2143), 30/III/1937 (ETH, HA Hs 975:2146) and 12/V/1938 (ETH, HA Hs 975:2147)).

28 To mention briefly some of Hertz’ wildest (unpublished) pipe dreams: Hertz mentions in a letter to Schlick dated 20/IV/1920 that he had been working on “irony” some time before 1920; in a letter to Bernays dated 30/III/1937 (ETH, HA Hs. 975:2146) Hertz mentions working on “the I (solipsism etc)” some time in the 1930s (an entry in Carnap’s diary, dated 22/X/1935 (Carnap 2022, p. 719), records that Hertz called Carnap a solipsist, which suggests that Hertz’ interest in solipsism might be connected to his engagement with Carnap). Both of these projects never came to anything and were eventually abandoned by Hertz but, it seems, not before Hertz had expended a significant amount of time and energy in their pursuit.

29 Though one might add that Carnap at the time had very strong and rather narrow views as to what constituted proper philosophical problems, as Felix Kaufmann noted in a letter to Carnap (held by the ASP under the signature RC 028-26-04, dated 19/II/1931), reproaching Carnap for his lack of sympathy for traditional epistemology, in which Kaufmann saw a tendency to overzealously “die Probleme mit den Scheinproblemen aus[zu]schalten”.

30 An overview of the contents of Hertz’ papers is available online: https://digital.library.pitt.edu/islandora/object/pitt%253AUS-PPiU-asp199401 (checked 29/X/2022). The author was able to spend time in Pittsburgh to study Hertz’ papers in detail, and produce a more detailed overview of their contents, thanks to an Archival Fellowship from the department of the History of Science at the University of Pittsburgh in the summer of 2023.

31 Hilbert 1935a, p. 148; although Hilbert does not explicitly mention completeness as a goal here (a testament to Hilbert’s lesser interest in the issue, perhaps), it is arguably implicit in his description of axioms as sentence “which are alone sufficient to erect from them according to logical principles *the entire structure* of a discipline” (Hilbert 1935a p. 147, emphasis mine).

32 Hertz 1921, p. 154; Hertz 1922, p. 246 opens with a similar passage. It is worth noting that this problem, and in particular the task of extracting a minimal independent axiom system from a given non-independent system, was suggested already in Hilbert’s 1900 address : “Bei näherer Betrachtung entsteht die Frage, *ob etwa gewisse Aussagen einzelner Axiome sich unter einander bedingen und ob nicht somit die Axiome noch gemeinsame Bestandteile enthalten, die man beseitigen muß, wenn man zu einem System von Axiomen gelangen will, die völlig von einander unabhängig sind*.” (Hilbert 1935c, p. 300)

33 Indeed, Tennant 2015, p. 385, commenting on Gentzen’s first article, where Gentzen adopts Hertz’ terminology, writes that in it what Tennant takes as a matter of course to be sequents are “unhelpfully called *Sätze*”, and elaborates that this is unhelpful precisely because of the confusion with the now “usual reading of *Sätze*” as a label for the *components of* sequents.

34 Nowadays, sequents are usually described in the way Gentzen 1935a, p. 180 already described them, as having as components *formulas*, not “elements” of an indefinite nature, which is how Hertz speaks of “Sätze” (e.g. in the official definition cited in footnote 43 or in the introductory remarks to his 1921 presentation, where Hertz calls *a* and *b* the “so verbundenen Elemente [eines Satzes a→b]” (Hertz 1921, p. 154). Since Hertz considers sentences (in his sense) as constructed out of abstract elements, Hertz—unlike Gentzen—does not regard them as having an internal logical complexity—such as the connective- or quantifier-structure of formulas—that is relevant to the deductive relations of the sentence/sequent itself.

35 Hertz 1922, p. 247; Hertz’ citation reads “[Whitehead-Russell 1 (1900), S. 15]”, which cannot be right. The reference is actually to Russell (1937), referred to as “Whitehead-Russell 1” perhaps because in the preface Russell 1937, p. xv portrays it as preparing a “Volume II” where “all its propositions [...] will be established by strict symbolic reasoning”, i.e. Whitehead/Russell’s *Principia Mathematica* I–III.

36 Gentzen later describes his sequent calculus as “logistical” (Gentzen 1935a, p. 190), and associates this label explicitly with the systems of “Russell, Hilbert, Heyting” (see: Gentzen 1935a, p. 184). Gentzen presumably means to highlight that there are no temporary assumptions in the sequent calculus: Every step is an assertion (of a conditional). In this way, though Gentzen does not mention Russell’s notion of a formal implication, the sequent calculus resembles Russell’s definition of “pure mathematics” (see: Russell 1937, p. 5) as consisting exclusively of assertions of formal implications.

37 Schröder-Heiste 2002, pp. 247 sq. observes in the same vein, first, that “[s]tructural rules may be looked upon as axiomatizing a consequence relation” and, second (on p. 248), that “structural systems [which he takes Hertz to have formulated, see p. 247] may be considered as providing a general framework of consequence”.

38 This character of Hertz’ framework, that its rules are not like rules of inference in a natural deduction system, is what I take scholars who see Hertz as a pioneer of “structural reasoning”, such as Schröder-Heister 2002 and Moriconi 2015, to be emphasising.

39 This can be regarded as the “positive heuristic” (in the sense of Lakatos) guiding Hilbert’s proof-theoretic work, its “research policy, or order of research” (see: Lakatos 1978, p. 50); it fits especially well another description Lakatos provides Lakatos 1978, p. 52, namely, it determines “[w]hich problems scientists working in powerful research programmes rationally choose”.

40 This provides a clear sense in which each iteration of Hilbert’s proof theory “constitutes a theoretically progressive problemshift”, as Lakatos (1978, p. 33) says: “each theory having at least as much content as the unrefuted content of its predecessor”—the axiom systems Hilbert considers are obtained by expanding the language and accepting additional axioms—and “each new theory has some excess empirical content over its predecessor” insofar as it succeeds in establishing the consistency of a larger portion of number theory.

41 Whilst I largely agree with the high regard for “the originality of Hertz’ vision” Arndt 2019, p. 270 expresses, I do think (contrary to Arndt 2019, p. 279) that it is possible to “connect [Hertz’ investigations] to the logical literature of his time”, in particular to Hilbert 1905.

42 The remark is “odd” mainly because of the conception of proofs it suggests: that proofs should be conceived as *sets*, i.e. as having no intrinsic order, contrary to the now standard conception, according to which proofs are (intrinsically ordered) *sequences* of formulas. (This conception was, in fact, introduced by Hilbert himself, first in print in Hilbert 1935d, p. 169, where a “Beweis” is defined as “eine Figure, die uns als solche anschaulich vorliegen muss”, which is defined (on p. 166) as “untereinander stehende Zeilen”; since Hilbert requires here that a proof must “be given for inspection” its lines must be given in a definite order; in lectures Hilbert 2013, p. 495 Hilbert defined a proof explicitly as “eine Figure bestehend aus Formeln, die in solcher Weise aufeinander folgen, dass [...]”.)

43 What Hilbert actually says is: “Der Inbegriff zweier Aussagen *A*, *B*, in Zeichen A|B [...] heißt ebenfalls eine Aussage und zwar heißt *A* dann die *Voraussetzung*, *B* die *Behauptung*. Voraussetzung und Behauptung können selbst wiederum aus mehreren Aussagen $A_1$, $A_2$ bez. $B_1$, $B_2$, $B_3$ usw., bestehen[,] in Zeichen: $A_1\text { u. }A_2\vert B_1\text { o. }B_2\text { o. }B_3$ [...].” (Hilbert 1905, p. 177; Hilbert does not consider the possibility of disjunctive antecedents, so there is no need for an explicit notation for the connective between $A_1, A_2$, since there is only one option). Besides the superficial similarity that both Hilbert and Hertz use the first letters of the alphabet for components of Aussagen/Sätze, in his official definition of sentences Hertz, like Hilbert, speaks of a sentence as an “Inbegriff eines Komplexes, der nur aus einem einzigen Element bestehen kann und *antecedens* heißt, und eines Elementes, das *succedens* heißt” (Hertz 1922, p. 249).

44 Hertz numbers all theorems and definitions in his article consecutively. Following a suggestion made by Klev 2018, p. 2 fn. 3 for Dedekind’s similarly structured *Was sind und was sollen die Zahlen?*, I will use the word “article” (in sense I.4 of the *OED* (https://doi.org/10.1093/OED/9634057113, accessed 9/IV/2026), which gives one example of this usage from the *Annals of Mathematics* 1927, or sense 3 of the *Digitale Wörterbuch der deutschen Sprache* (https://www.dwds.de/wb/Artikel, accessed 9/IV/2026)) for these numbered portions of text (since, like Dedekind, Hertz uses the paragraph symbol “§” for larger sections, and “section” usually portions of text longer than a single paragraph). Speaking of Dedekind: It is worth noting that the word “Kette” serves as a central technical term for Hertz 1922, Dedekind 1965, art. 37 though Hertz defines chains ultimately (by way of art. 19) unlike Dedekind defines them, Hertz still defines them in Dedekindian style, so to speak; namely, in terms of a partitioning analogue to a Dedekindian “Cut” of elements into two classes (art. 17) according to whether a sentence connects them or not. What is more, within the context of what Hertz calls a “closed system” of sentences (Hertz 1922, art. 5) (i.e. one which contains every sentence derivable from its sentences), in whose independent axiomatisation Hertz is ultimately interested, such chains are, like Dedekind’s chains, (effecitvely) the range of the transitive closure of their main binary relation—i.e. Dedekind’s ϕ or Hertz’ abstract binary dependence relation →, which his sentences express—applied to elements of the given set: In Hertz’ terms, every sentence in a closed system that belongs to any chain belongs to a maximal chain (art. 32), which is itself closed (art. 29).

45 Franks 2023, p. 99fn6 calls Gentzen’s Cut rule “a slight modification” of this rule; Franks 2023, p. 280 remarks furthermore that both Gentzen’s Cut and Hertz’ Syllogism rule “share with the classical syllogistic forms an intermediate [so-called “middle] term[”] that appears in each premise but is missing from the conclusion”.

46 The difference between Hilbert’s conception of proofs and Hertz’ conception of inference systems is neatly illustrated by their choice of metaphor: Hilbert, in the very passage where he introduces his lemma, calls the general kind of things of which proofs are a specific sort a (linear) “Formelkette”; Hertz, by contrast, speaks of displaying logical dependencies “Form eines Stammbaums” (Hertz 1923a p. 77, as Gentzen does later: Gentzen 1935a, p. 181).

47 Schröder-Heister 2002, p. 251 fn. 14 also notes the affinity between Hertz’ result about proofs and inference systems, and Hilbert’s Lemma, which I’ve called “Disentanglement Lemma” because it disentangles the threads (in Hilbert’s technical sense) of a proof. Sieg 2013, p. 176 fn. 38 even claims that “Gentzen learned about the tree representation of proofs” from Hilbert’s Lemma, as presented to him in Hilbert’s 1929 set-theory lecture course (see footnote 5.2). It seems more likely, however, that Gentzen learned about proof trees from Hertz’ work, which contains them explicitly, not just implicitly, as Hilbert’s Lemma does.

48 A similar preliminary modification of a whole deduction, to obtain a deduction where “every sequent in the deduction shall be used to obtain at most *one* further sequence”, obtained by “proceeding from back to front, to write down sequences used multiple time each as often as needed, along with the sequences used to deduce them” is outline also later by Gentzen as part of his consistency proof, see Gentzen^1936^, p. 542.

49 The word “issue” is here to be understood in the sense of “child”—as still standardly used in the genealogies of royal families. The German word Hilbert uses, “Kömmling”, is peculiar. The Brothers Grimm note in their German dictionary only that it was “used only once by Goethe in Faust 2, act 5” (in the sense of somebody who arrives; cf: https://www.woerterbuchnetz.de/DWB?lemid=K10390 (accessed 27/XI/2024)). That “Kömmling” might be rendered into English as “issue” was suggested to me by Hilbert’s use of “Abkömmlinge” in the sense of “children” in his lecture on the infinite (see Hilbert 2013, p. 722).

50 In the official lecture notes prepared by Bernays, the exposition of these techniques takes up little more than a page (Hilbert 2013, p. 506-508); in the surviving student notes, it takes up only a single paragraph (of the session dated 14/XII/1922, see Hilbert 2013, p. 615). A reviewer has suggested taking the brief exposition of disentanglement in the official lecture notes for 1921/22 as evidence that Hilbert arrived at these ideas independently. I think the situation is less clear. As the editors note (see: Hilbert 2013, p. 419), there are “important differences” between these official lecture notes and the actual lectures as recorded independently by a student—in particular on this point: The student notes do not record any discussing of disentanglement in the academic year 1921/22, only in 1922/23 (cited above); in fact, the portion of the official notes where disentanglement is covered are paginated separately from the rest of the notes, which suggests that the treatment of disentanglement was not part of these notes originally, but rather added subsequently. That Hilbert (1935d) does not contain such considerations either—they appear in print for the first time in Hilbert 1935b, p. 184—suggests also that they were worked out only in 1922/23, at the earliest in the summer of 1922 in preparation of the course in the fall, at which point Bernays would have read Hertz (1923a) in manuscript (indeed, Hertz 1923a, p. 81 expressly thanks Bernays for conversations on “multiple occasions”), and even Hilbert himself may have seen the manuscript in his capacity as editor of the *Mathematische Annalen*. All this does not provide positive evidence *that* Hilbert or Bernays took their cues for thinking about disentanglement from Hertz, but I think I can say with some confidence that this cannot be ruled out purely on chronological grounds.

51 A few letters from Hertz to Hilbert exist in Hilbert’s Nachlass held by the SUB Göttingen (their signature is “Cod. Ms. D. Hilbert 150”; the letters found amongst the Hertz’ Papers at the Archive of Scientific Philosophy in Pittsburgh under the signatures “PH 01-09-01” through “-04” are all merely photocopies of those found in Göttingen), but they concern only Hertz’ move back to Göttingen. No theoretical questions are discussed.

52 Arndt 2019, p. 270 seems to regard the fact that Hertz “was not influenced by the logical industry that was taking place next doors [sic]” as evidence for Hertz’ originality. I am not inclined to interpret the apparent lack of communication between Hertz and Hilbert in this way; and Hertz’ acknowledgments show that Bernays did have some influence on Hertz’ work.

53 It may be worth noting in this context that Max Born tells Moritz Schlick in a letter dated 16/IV/1931 that neither the mathematicians, nor the physicists in Göttingen—although Born names no names—encouraged Hertz in his work, which was perceived as straying too freely across disciplinary boundaries. Though this would hardly have deterred Hilbert, who not only freely engaged in physics, logic and philosophy himself, but even wrote (in an unpublished note kept by the SUB Göttingen in a convolute of similar note papers under the signature “Cod. Ms. D. Hilbert 603”, where this note is found on fol. 20): “Die Natur kennt keine Vorurteile u. keine Vorbildung, wie die Fakultäten, keine Einteilung in Fächer!”

54 Another (minor) point of contact between Hert & Hilbert is the notion of a “thread” in a proof: The German word “Faden” appears to have been used first in the lecture course of 1922/23 on 11/I/1923 (by Bernays), see Hilbert 2013, p. 619. (That “Faden” appears in quotation marks in Kneser’s notes suggests it was introduced as a new technical term at this point.) Substantially the same notion had been defined in Hertz 1923a, p. 86, though Hertz speaks of “Teilkette”—where “Kette” has a different sense than that discussed in footnote 44—instead of “Faden” (as Hertz himself noted in Hertz 1929b, p. 464 fn. 14).

55 The following consideration, by way of which Hilbert may have been led to the idea of adding ideal statements to theories independently of Hertz’ work, was suggested by a reviewer.

56 The reports discussed here incidentally corroborate a remark by von Plato, who claims that the idea of a sequent “was known well in Göttingen [in the 1920s]” because “Paul Hertz had written long papers on sequents that Bernays endorsed and got published in the *Mathematische Annalen*” (von Plato 2014, pp. 1178 sq.) As far as I can tell, what von Plato says about Bernays’ role in the propagation of Hertz’ work is pure conjecture; von Plato, in any case, points to no supporting evidence. (Indeed, whilst von Plato happens to be right about Hertz’ work being known in Göttingen—as I will go on to document here—von Plato himself points to no evidence in support of this claim, either.) I am not aware of any evidence that Bernays, in particular, played a crucial role in getting Hertz’ articles published, or in *initially* propagating Hertz’ work in Göttingen (von Plato may be extrapolating from Bernays’ *later* advocacy on Hertz’ behalf, e.g. in a letter to Szabo referred to in Plato 2017, p. 11).

57 The report reads (in full): “Gruppentheoretische Bemerkungen zum Problem der Axiomatik. Die überlegungen von *P. Hertz* lassen sich gruppentheoretisch fassen.” Paul Hertz’ son Rudolf recalls on p. 10 of the “Biographical Essay” referred to in footnote 7 that Emmy Noether became a close friend of the Hertz family in the early 1920s.

58 Hertz, in fact, uses the word “Gruppen” both in his first programmatic article (Hertz 1921, pp. 156, sq.) and in his first technical article (Hertz 1922, art. 54) (in connexion with ideal elements), though not recognisably in the technical sense of group theory.

59 The notes are found amongst Behmann’s papers, now held by the Staatsbibliothek Preußischer Kulturbesitz in Berlin (SBB) as Nachlass 335, in box 9 as item 33. They bear the title reported in the *JDM*: “Zum Problem der Axiomatik vom Standpunkt der Algebra der Logik”. The complete text of Behmann’s notes, which are of interest, I think, also beyond what they show about (engagement with) Hertz, as a document of the development of mathematical logic during the 1920s, is published here with the kind permission of the SBB as an appendix.

60 Von Plato 2009, p. 682 reports a similar remark from unpublished lecture notes where Bernays maintains in 1935 that, due to the availability of a decision procedure, “the performing of deductions becomes quite superfluous” in propositional logic, which was exactly Behmann’s reasoning in 1922 and Bernays may even (since he presumably attended the Hertz-Week) be echoing Behmann’s assessment—Behmann was, after all, the authority on the decision problem –, or expressing a consensus reached amongst the logicians in Göttingen.

61 Arndt 2019, pp. 279 sq. comes to a similar assessment of Hertz’ results (without knowing of Behmann’s presentation), writing about Hertz first article that “the first part of his investigations presented here is extremely limited in scope due to the restriction to singleton antecedents and the (correspondingly) very basic inference rule. [...] One might say that Paul Hertz creates the expectation in the reader that an investigation into matters logical is to follow and then presents something so unconventional and so full of original terminology and self-stated problems that merely arise within this system and do not appear to be of independent interest. In other words, whilst Hertz talks about logic, his problems and results appear to have nothing to do with what might at the time of publication have been | considered to be significant (especially for foundational questions).” This last sentence describes precisely the sentiment Behmann appears to have expressed in his presentation in 1922.

62 In fact, diagrams found in the student notes for Hilbert’s 1922/23 lecture course (see: Hertz 2013, p. 615,618) might represent another trace of Hertz’ influence on Hilbert’s proof theory: Whilst they do not resemble the proof trees Hertz uses in his technical articles, they do resemble certain diagrams Hertz included in his informal presentation of 1921 to visualise deductive dependence by means of nodes and arrows (see: Hertz 1921, 156), which at least Bernays (who, in 1922, presents the diagrams) probably attended (see footnote 14), and on which Behmann reflected at some length in his response to Hertz’ presentation.

63 Although Hertz 1921, p. 157 does contain one diagram, fig. 5a, representing sentences as points and using plane figures to indicate an unspecified deductive relationship amongst them.

64 Whilst it is far from obvious how to generalise Behmann’s Cube-diagram for more than 3 elements (since the obvious generalisation would lead to polytopes in *n* dimensions (where *n* is the number of elements considered) with 2*n* faces (one for each element, one for its negation) such that in each vertex *n* faces meet, which, however, whatever its precise shape, won’t help harness geometrical intuitions for (meta-)logical purposes); this method for diagrammatically checking (in-)dependence of axioms (in a sentential logic setting), by contrast, lends itself to extension: for *n* elements (plain) plane grid with $2^n$ positions is needed.

65 This result is partial but, as Gentzen later showed (in: Gentzen 1932, p. 329), also as good as it gets (an extensive reconstruction of the results of Gentzen’s first article is offered by Moriconi 2015, §§ 2-4; Franks 2010, pp. 366–369 also discusses the article (in less detail)): Insofar as the leading question of Hertz’ research project has an answer, it is negative: No unique independent axiom systems exist in most cases of (interesting) systems of sentences. Indeed, already within sentential logic—and certainly in more sophisticated settings, such as first-order logic—there are endless examples of non-trivially inter-deducible sentences. This negative result may well have been the reason why nobody pursued Hertz’ project further.

66 In fact, the informal outline of the notion of a cycle in Hertz 1923a, p. 78 foreshadows the requirement “that [sentences of a cycle] be neither *trivial* nor *abundant*”. But then the official definition of “cycle” in art. 89 expressly excludes only tautological sentences. It is possible that this is simply a slip of the pen, and Hertz should have written “trivial” instead of “tautological” in his official definition, in accordance with the informal outline. But I think the definition of “abundant” (in art. 57) along with that of a deductively closed system of sentences (in art. 24) as including all tautological sentences entails that, when considering a system of sentences which is deductively closed, all trivial sentences are abundant (given the tautological sentences). Indeed, Hertz 1923a, p. 97 explicitly asserts the contrapositive: “Any non-tautological [and] non-abundant sentence is not trivial”, hence every trivial sentence is either tautological (if it has only a single antecedent) or (if it has multiple antecedents) abundant.

67 Studying the conditions under which deductions reliably indicate genuine deductive dependence can be regarded as the positive heuristic of Hertz’ proof-theoretic work. In this Hertz’ work differs fundamentally from Hilbert’s (as described in footnote 39). Hertz’ and Hilbert’s work can thus be regarded as contributing to a shared research programme of proof theory, united by a shared “hard core” (see p. 2), but distinguished one form the other by their respectively different positive heuristics, which are, as Lakatos acknowledges, “in general more flexible than negative heuristics” (Lakatos 1978, p. 51) within a single research programme.

68 The following is the definition (in art. 75) of what Hertz calls “an inabundant inference system of the second kind”, in contrast to a first kind of inabundant inference system defined in Definition 74, which, however, plays no role in establishing Hertz’ main results. The usage of “(in)abundant” for inference systems is distinct from the usage of “(in)abundant” for individual sentences, though the latter features in the definition of the former; but the conditions for being an inabundant inference system are more stringent than merely precluding any sentence in the inference system being an abundant sentence.

69 Hertz defines immediate inference (in art. 13) as an inference from a sentence to any sentence whose antecedent includes the antecedent of the former alongside additional elements (see: Hertz 1923a, p.82); officially, immediate inference is defined in terms of “containment”, which is defined on the same page in art. 9. The same rule Hertz calls “immediate inference” Gentzen calls “thinning of the antecedent”; the kinship of Hertz’ Immediate Inference and Gentzen’s Thinning of the Antecedent was noted also by Bernays 1965, p. 6, and in fact Gentzen 1932, p. 331 already noted when first introducing his preferred label for the rule that it is “called immediate inference by Hertz”. Note that any conclusion of an application of Immediate Inference is abundant relative to the premiss. So condition (c) is actually entailed by (a): Since an inference system must consist only of sentences taken from the underlying system of sentences, a sentence obtained from another by immediate inference would generally be abundant relative to the underlying system. So any application of immediate inference is in effect excluded by condition (a).

70 Note, by the way, the similarity of this to Hilbert’s main result that every numerical sentence, if it is provable at all (within a given axiom system), can be proved by means of a purely numerical proof. But note also the difference: Hilbert’s result concerns *the sort of formulas* used in the deduction, whilst Hertz imposes conditions also on the overall course of the deduction by proscribing the use of the immediate inference rule.

71 Calling them “top sentences” presumably harks back to Hertz’ relation $\textsf{Z}$ (discussed on p. 14), which relates premisses of (later) applications of inference rules to conclusions of (earlier) applications of inference rules: In this (non-linear) order amongst lines of an inference system, all (but not only) major premisses of a normal proof in Hertz’ sense are maxima

72 Hertz’ terminology is, as Hertz acknowledges in fn. 27, traditional (one finds it, e.g. in Drobisch 1936, §87; Schröder-Heister 2002, p. 253 also notes the affinity between Hertz’ results and “the traditional distinction between Aristotelian and Gocklenian chain syllogisms”, without explicitly citing any source in particular for this distinction.)

73 Gentzen also establishes his normal form results in the same way Hertz does (by means of an inductive argument on the construction of deductions, as was noted by Schröder-Heister 2002, p. 254, who remarks: “It is obvious that Hertz’s proof [of his normal form result] could provide considerable inspiration to Gentzen, who later made extensive use of such methods.”

74 Continuing in the Lakatosian vein, one might argue—if one accepts the proposal by Hallett 1979, p. 6 that mathematical progress in the sense relevant to Lakatos’ methodology of research programmes consists in new results being “applied in unexpected ways, to solve problems they were not originally designed to cover”—that only at this does proof theory lead to any palpable mathematical progress, by yielding not only the consistency results it was designed to deliver, but, in addition, certain independence results. Schröder-Heister 2002, p. 253 notes perhaps this same evolution of proof theory by learning how to appreciate results its inventors did not set out to establish, when he remarks: “For both Hertz and Gentzen, these results played an auxiliary role in their treatment of axiomatizability. From a more modern point of view they contain fundamental conceptual insights.”

75 Moriconi 2015, p. 677fn28 notes that the “Kettenschluss”-rule Gentzen introduces to his system in 1936 is “[s]trongly cognate to the Syllogism rule of P. Hertz” . On Gentzen 1936, p. 543, Gentzen details how “the disregarded *inference rules* admit of replacement by the new base sequents, which take their place, and recourse to *Kettenschluß*”. In this context Gentzen stresses the feature I have suggested (on p. 31) is key to a fruitful normal-form result (in proof theory): that “the new *concept of deduction* is thus not narrower than the *old one*, i.e. everything deducible is deducible by normal-form deductions.”.

76 This explains why Gentzen in 1936 includes, as a preliminary, a result like Hilbert’s Disentanglement Lemma (see footnote 48): Because inferences can be safely juxtaposed only if no intermingling threads run between them.

77 This procedure is set out in Gentzen 1936, §. 14.2 4 . It bears out a programmatic remark Hilbert 2013, p. 370 made in 1920: that the use of induction “only shortens the proofs”.

78 This is a notable difference between Gentzen’s first and second “Widerspruchsbeweis”: In the first (of 1936), Gentzen assigns ordinals only to “any given deduction (in the sense of 14.1)” (Gentzen 1936, p. 550), i.e. only to deductions in Kettenschluss normal form (introduced in §14.1). By contrast, in the Gentzen (1936), ordinals are assigned to “an arbitrary” (i.e. not necessarily normalised) deduction (Gentzen 1938, p. 39.

79 It is worth noting that Gentzen 1932, p. 344 uses this property (which he formulates on p. 343 as Lemma No. 6) to infer that certain sentences *cannot* be deduced from certain axioms since all axioms have a certain antecedent, but the sentences in question do not: so those sentences cannot be deduced from these axioms by means of a normal proof, hence not at all.

80 The main aim of Arndt’s remarkable article Arndt (2019) is to highlight another field where Hertz had considerable impact: the theory of directed graphs, where Hertz’ pioneering work, although initially generously acknowledged (by Dénes Kőnig), as Arndt documents (see: Arndt 2019, sec.5.2), has likewise gone unappreciated. Arndt 2019, p. 288 suggests that an inexplicable omission in the first English language textbook on graph theory “disconnected the original work of Paul Hertz from the subsequent literature on graph theory”.

81 Indeed, the pinnacle of proof-theoretic achievement obtained by Hilbert’s own circle of students and collaborators, the methods of ε-substitution, is nothing other than a normal-form result for complex terms in an artificial language containing the (“*”transfinite) ε-operator, which (ultimately) transforms all terms into the “*”especially perspicuous, indeed, canonical form of terms in an arithmetical language, namely numerals.
